# AI‐Assisted Design and Evaluation of SLM‐Ti64 Implants for Enhanced Bone Regeneration

**DOI:** 10.1002/adhm.202503154

**Published:** 2025-09-04

**Authors:** Muhammad Usama Zaheer, Muhammad Hassan Razzaq, Mehmet Fatih Aycan, Yogendra Kumar Mishra

**Affiliations:** ^1^ Smart Materials NanoSYD, Mads Clausen Institute University of Southern Denmark Alsion 2 Sønderborg DK‐6400 Denmark; ^2^ Department of Mechanical Engineering Institute for Graduate School of Natural and Applied Sciences Gazi University Ankara 06570 Türkiye; ^3^ Additive Manufacturing Technologies Application and Research Center‐EKTAM Gazi University Ankara 06570 Türkiye

**Keywords:** additive manufacturing, artificial intelligence (AI), biomechanical simulation, orthopedic implants, tissue differentiation

## Abstract

This study presents a comprehensive framework combining Selective Laser Melting (SLM) of Titanium (Ti64) alloys, finite element simulation, and artificial intelligence (AI) to advance orthopedic implants' design and predictive evaluation. Dense Ti64 specimens are fabricated using ten distinct SLM parameter sets to explore the effects of volumetric energy density (VED) on mechanical behavior, porosity distribution, and microstructural integrity. Optimal VED ranges are identified to balance defect minimization and mechanical performance, with porosity levels strongly influencing tensile strength and Young's modulus. These material properties are then integrated into a mechanobiological bone healing model to simulate fracture repair in tibial mid‐diaphyseal regions under intramedullary fixation. A mechano‐regulation algorithm incorporating deviatoric strain, fluid velocity, and pore pressure guided tissue differentiation and callus evolution over a 4‐month healing period. Variations in implant stiffness—achievable via solid and lattice Ti64 configurations demonstrate distinct influences on endochondral ossification, external callus growth, and overall biomechanical recovery. AI‐based regression models are trained on simulation outcomes to accelerate evaluation and enable patient‐specific predictions to forecast healing trajectories without repeated finite element analyses. This AI‐driven framework facilitates real‐time prediction of healing outcomes based on implant design, supporting the development of adaptive and optimized orthopedic implants for clinical translation.

## Graphical abstract

AI‐driven simulations of biological healing, combining biomechanical modeling and machine learning, enable personalized orthopedic treatments. By decoding healing patterns influenced by implants and patient‐specific factors, this approach advances fracture repair understanding, optimizes implant design, and supports precision medicine and sustainable healthcare.

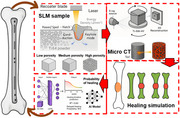

## 1 Introduction

Biomedical implants have transformed modern medicine by restoring function and alleviating pain in patients suffering from degenerative diseases, trauma, and skeletal deficiencies.^[^ ^1^ ^]^ The growing incidence of conditions like osteoporosis and arthritis has driven the demand for advanced implant technologies that can support or replace damaged tissues.^[^ ^1^, ^2^ ^]^ Orthopedic and dental implants play a vital role in restoring mobility and function, yet their long‐term success depends not only on surgical accuracy but also on mechanical compatibility, integration with bone, and durability under physiological loads.^[^ ^3^ ^]^ One of the main challenges in implant design is the mismatch in elastic modulus between implant materials and bone, which can cause stress shielding. In this phenomenon, a stiffer implant absorbs most of the mechanical load, reducing bone remodeling and leading to bone loss. Low‐modulus materials and porous structures can reduce this mismatch, enhance osseointegration, and support natural bone regeneration.^[^ ^4^ ^]^ Implant performance is also influenced by design and load transfer dynamics; while compressive stress supports bone formation, shear forces can disrupt integration. Porous structures increase surface area and permeability, improving both mechanical interlocking and nutrient diffusion—critical factors for bone healing.^[^ ^5^ ^]^ Furthermore, implant materials' fatigue strength and wear resistance are paramount for long‐term functionality, particularly in load‐bearing applications. Metallic implants, such as those made from titanium and its alloys, are widely used due to their superior strength, biocompatibility, and corrosion resistance. However, emerging biodegradable materials, such as magnesium‐based alloys, are gaining attention for their ability to provide temporary support while gradually degrading in vivo, eliminating the need for secondary surgeries. Striking a balance between degradation rates and mechanical stability remains a critical focus in the design of these materials.^[^ ^6^ ^]^

**Figure 1:**
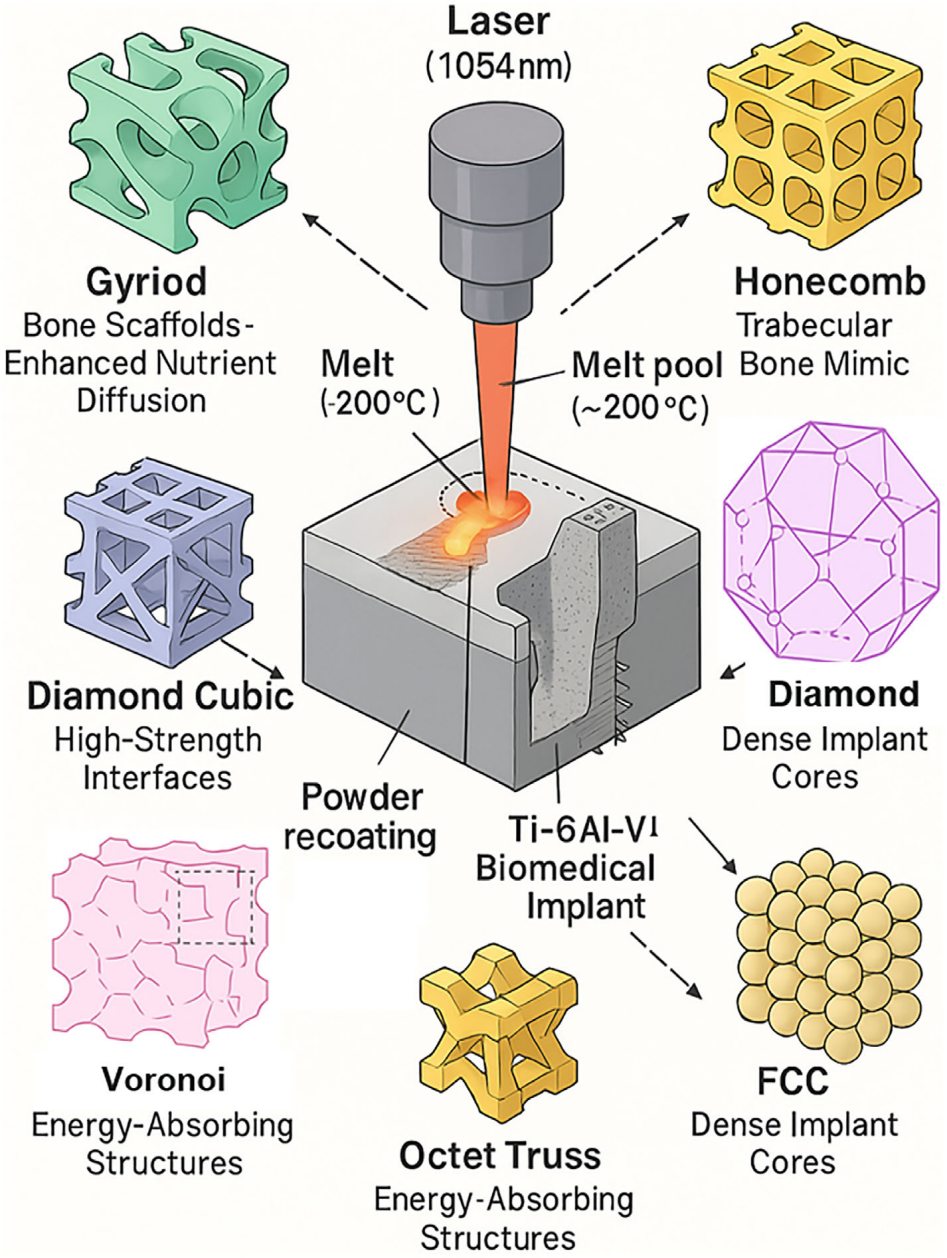
Illustration of the SLM process and representative lattice structures. The image shows the laser‐based melting of powder bed material to form complex 3D lattice geometries, demonstrating the versatility of SLM in fabricating intricate designs with tailored mechanical properties for biomedical and engineering applications.

Additive manufacturing (AM), particularly selective laser melting (SLM)^[^ ^7^ ^]^ offers new opportunities to design and fabricate implants with tailored mechanical and biological properties.^[^ ^8^ ^]^ Unlike traditional manufacturing, SLM allows fabrication of complex, patient‐specific geometries directly from digital models. Ti‐6Al‐4 V (Ti64), a widely used alloy for implants, benefits from the geometric flexibility and microstructural control enabled by SLM.^[^ ^9^ ^]^ One of the most significant advantages of SLM in the context of biomedical implants is its capacity to address the issue of stress shielding through controlled porosity.^[^ ^10^ ^]^ These porous structures also promote bone ingrowth and vascularization, which are essential for long‐term stability.^[^ ^11^ ^]^ Lattice structures have emerged as a powerful design strategy for orthopedic implants. Mimicking the hierarchical structure of bone, they combine mechanical support with biological functionality.^[^ ^12^ ^]^ **Figure** **1** shows different lattice structures that are possible for implant manufacturing using SLM.

Among the most utilized designs are strut‐based geometries and triply periodic minimal surfaces (TPMS), each offering unique advantages.^[^ ^13^ ^]^ Strut‐based lattices like body centered cubic (BCC), face centered cubic (FCC), and octet‐truss are popular for their design simplicity and ease of manufacturing. BCC lattices offer low density and reduced stiffness, helping prevent stress shielding, while variants like BCC‐Z add diagonal struts for greater stability. FCC and FCC‐Z provide higher strength and structural integrity, suitable for load‐bearing use. Octet‐truss lattices, being stretch‐dominated, deliver excellent stiffness and strength, ideal for implants under high mechanical loads.^[^ ^12^, ^14^ ^]^ Each has unique characteristics: BCC and BCC‐Z offer compliance; FCC‐Z and octet‐truss provide strength for load‐bearing scenarios; gyroid and Schwarz diamond structures support stress distribution, vascularization, and tissue ingrowth. **Table** **1** summarizes the key properties and SLM suitability of these designs.^[^ ^14^, ^15^ ^]^ Lattice properties can be tuned by adjusting cell size, strut thickness, and relative density, allowing implants to mimic natural bone mechanics. Elastic modulus can be matched to cortical or trabecular bone to reduce stress shielding. Stretch‐dominated designs like octet‐truss and gyroid offer high strength and stiffness, while bending‐dominated structures like BCC provide more compliance for low‐stress regions. Their high strength‐to‐weight ratios ensure lightweight yet durable implants, and the porous architecture promotes bone regeneration and integration.^[^ ^14^, ^15^ ^]^ Design parameters like cell size, strut thickness, and density can be tuned to adjust stiffness and permeability. Open pores (100–600 µm) support osseointegration, while high surface area enhances cellular attachment. These characteristics make lattice‐based implants lighter, biologically active, and mechanically compatible with bone.^[^ ^12^, ^14^, ^15^ ^]^ Table 1 depicts the overview of lattice structures used in bone implants.

**Table 1:** Overview of lattice structures used in bone implants, their key properties, applications, and SLM suitability.

Lattice Type	Key Properties	Applications	SLM Suitability	Refs.
BCC	Low density, bending‐dominated, suitable for reducing stiffness and preventing stress shielding	Regions requiring lower stiffness to minimize stress shielding	Easily manufacturable with good dimensional accuracy	[12, 15]
BCC‐Z	Enhanced stability with diagonal struts, maintains low weight, higher strength than BCC	Intermediate load‐bearing implants with improved strength	Requires moderate optimization for structural stability	[15]
FCC	High stability and strength, stretch‐dominated, better for load‐bearing applications	High‐stability implants in regions with significant load	Precision fabrication with predictable mechanical performance	[14]
FCC‐Z	Added diagonal struts for improved load‐bearing capacity and stiffness	Heavy load‐bearing implants with superior mechanical properties	Optimized for high‐strength applications with SLM adjustments	[14]
Octet‐Truss	High stiffness and strength, stretch‐dominated, excellent for high‐load applications	Critical load‐bearing scenarios in orthopedic applications	Ideal for strength‐critical applications, it benefits from SLM process control	[12, 15]
Gyroid	High surface area‐to‐volume ratio, continuous porosity, excellent nutrient diffusion, and osseointegration	Highly vascular regions requiring osseointegration and nutrient diffusion	High manufacturability with SLM, excellent for complex designs	[12]
Schwarz Diamond	High mechanical strength, uniform stress distribution, suitable for load‐bearing applications	Load‐bearing implants with high mechanical reliability	Precision fabrication achievable with SLM for load distribution	[15]
Neovius	Moderate strength, high permeability, ideal for metabolically active regions with fluid transport needs	Bone scaffolds in highly vascularized and metabolically active regions	Suitable for SLM with controlled porosity and fluid transport design	[15]

SLM also enables patient‐specific customization using imaging data (e.g., CT scans), ensuring better anatomical fit and reducing surgical complications. Process parameters like laser power and scan speed can be optimized to tailor microstructure, fatigue strength, and wear resistance.^[^ ^16^ ^]^ SLM allows precise control over Ti64 microstructure and mechanical properties by tuning process parameters like laser energy and scan speed. This enables the production of refined microstructures with improved fatigue strength and wear resistance, ideal for load‐bearing applications. Additionally, bioactive coatings can be integrated during printing to enhance implant performance.^[^ ^9^, ^17^ ^]^

To better predict healing outcomes, mechanobiological models simulate bone regeneration over time by integrating mechanical loading, biological signaling, and tissue differentiation. These models, typically based on finite element analysis (FEA), provide valuable insights but are computationally demanding and require expert knowledge.^[^ ^13^, ^18^ ^]^ To overcome this, artificial intelligence (AI) offers an efficient alternative. By learning from existing FEA data, AI models can predict bone healing patterns without rerunning simulations, making predictive tools more accessible to clinicians.^[^ ^19^ ^]^ One of the key advantages of integrating AI into mechanobiological predictive models is the ability to provide real‐time assessments of implant performance under varying clinical conditions. For instance, AI can predict how changes in implant design, material properties, or mechanical loading might impact the healing process, offering immediate feedback on the suitability of specific treatment strategies. Additionally, these models can account for patient‐specific factors such as age, bone density, and fracture geometry, further personalizing predictions. By eliminating the need for repeated FEA simulations, AI‐enhanced models allow clinicians to explore multiple scenarios rapidly, optimizing implant designs and surgical interventions to achieve better outcomes.^[^ ^18^, ^20^ ^]^

AI enables rapid scenario analysis by incorporating implant design, loading conditions, and patient‐specific variables (e.g., age, bone density), allowing real‐time prediction of implant performance and personalized treatment optimization. Complementing this, SLM can precisely fabricate AI‐recommended designs such as optimized gyroid structures, ensuring accurate translation from prediction to production.^[^ ^19^, ^21^ ^]^ Moreover, AI‐enhanced models can monitor post‐operative healing by comparing clinical data to predicted healing trajectories. Early detection of deviations enables timely interventions, such as adjusting rehabilitation protocols to improve outcomes.^[^ ^18^, ^19^ ^]^

Artificial intelligence (AI) and machine learning (ML) are transforming research and industry, driving major advances across healthcare, finance, manufacturing, and engineering.^[^ ^22^ ^]^ AI enables machines to perform tasks requiring human intelligence, such as pattern recognition and decision‐making. ML, a subset of AI, allows systems to learn from data and improve performance without explicit programming.^[^ ^23^ ^]^ The integration of AI and ML in biomedical engineering has advanced medical imaging, disease diagnosis, drug discovery, and personalized treatment. This progress is driven by increased computational power, abundant data, and deep learning breakthroughs. Researchers now widely use AI to tackle complex challenges, from disease detection to designing optimized biomedical materials.^[^ ^24^ ^]^ In our work, AI accelerates the process of analyzing bone healing simulations and enables more efficient implant design by predicting patient‐specific healing outcomes in real‐time.

Machine learning algorithms fall into three main categories: supervised, unsupervised, and reinforcement learning. Supervised learning uses labeled data to learn input‐output relationships, making it ideal for medical diagnostics and predictive modeling.^[^ ^25^ ^]^ Unsupervised learning, in contrast, identifies hidden patterns in unlabeled data, often used for genetic classification and anomaly detection in biomedical research.^[^ ^26^ ^]^ Reinforcement learning (RL) enables decision‐making through rewards and penalties, playing a significant role in robotic surgery and adaptive healthcare systems. Similarly, machine learning problems can also be subdivided into classification and regression tasks.^[^ ^27^ ^]^ Classification assigns data to categories, while regression predicts continuous outcomes. Our study, focused on forecasting bone healing from lattice and mechanical properties, is a supervised regression task, estimating healing efficiency, tissue differentiation, and implant stability.^[^ ^28^ ^]^ This predictive capability allows for precise and adaptive treatment strategies, enhancing patient recovery by optimizing implant designs and rehabilitation protocols.

Several regression algorithms are used in ML, each with unique strengths. Linear and polynomial regression models represent simple and nonlinear trends, respectively. Decision trees and random forests handle complex, non‐linear data through recursive splitting, while Support Vector Regression (SVR) is well‐suited for small datasets and complex relationships by mapping data to higher dimensions.^[^ ^29^ ^]^ Artificial Neural Networks (ANNs) mimic the brain's structure to learn complex patterns, making them well‐suited for modeling biomechanical interactions. In our study, ANNs enable accurate, patient‐specific healing predictions by learning from simulated and experimental data, reducing the need for repeated finite element simulations.^[^ ^30^ ^]^ All these regression techniques, including ANNs, will be implemented in our work to ensure comprehensive and comparative analysis.

This study aims to optimize bone fracture healing through a combined approach of experiments, computational modeling, and AI. Ti64 samples were fabricated via SLM using varied parameters—laser power, scanning speed, and hatch spacing—to produce distinct mechanical properties. These variations enabled exploration of different microstructures, including porosity, modulus, and tensile strength, essential for orthopedic implant performance. A bone healing model was developed in Simulia Abaqus using realistic fracture geometry and a mechanoregulation algorithm to simulate healing over four months. The model incorporated biological and mechanical factors such as interfragmentary strain, mechanical loading, and tissue differentiation to capture the healing stages from callus formation to remodeling.

To ensure realistic simulations, we used mechanical properties from literature‐reported lattice structures—gyroid, Schwarz diamond, and BCC—known for tunable behavior and high surface‐area‐to‐volume ratios. Simulations with varying stiffness, porosity, and strength generated a diverse dataset reflecting multiple implant configurations and healing responses. This dataset trained machine learning algorithms to build a predictive model for implant healing efficiency over time. The AI framework eliminates the need for repeated FEA, enabling real‐time evaluation of implant performance and providing clinicians with actionable insights for treatment planning.

## 2 Experimental Section

### 2.1 SLM Specimen Preparation Methodology

**Figure 2:**
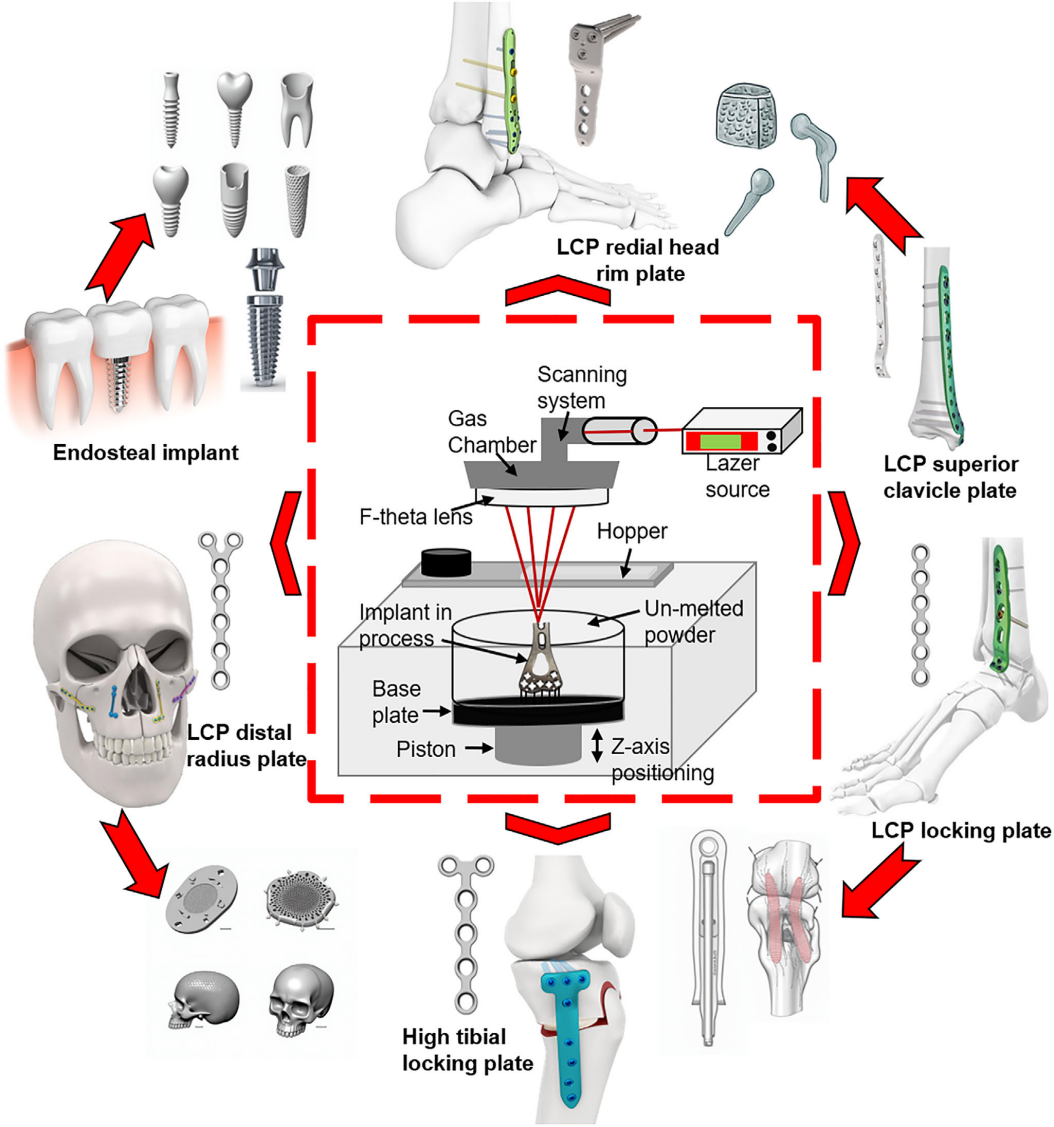
Illustration of additive manufacturing in biomedical implants, showcasing SLM for fabricating customized implants. The central schematic depicts the SLM process, where a high‐powered laser selectively melts Ti64 powder in a layer‐by‐layer manner to build implants with precise geometries. The surrounding images represent various biomedical implant applications, including dental endosteal implants, craniofacial fixation plates, orthopedic bone plates, and joint prosthetics. These implants are tailored for different anatomical locations, demonstrating the versatility and biocompatibility of SLM‐fabricated implants in patient‐specific treatments. The unmelted powder acts as support material during printing and is later removed, ensuring efficient material utilization.

Ti64 powder, extensively utilized in biomedical implant manufacturing (20–40 µm), was procured from General Electric GE USA. Dense tensile specimens of Ti64 alloy were fabricated on an Ermaksan ENAVISION 250 SLM machine at the Additive Manufacturing Technology Application and Research Center (EKTAM), Gazi University, Ankara, Turkey. The specimens were manufactured according to ASTM E8 standard to investigate the relationship between Volumetric Energy Density (VED) and mechanical performance. VED, a critical parameter in SLM, quantifies the energy input per unit volume and directly influences microstructure, density, and mechanical properties. Optimal VED ensured complete melting, reduced porosity, and strong layer bonding, while deviations can lead to defects such as incomplete melting, porosity, or keyholing. By precisely controlling VED, manufacturers can tailor microstructure, tensile strength, and fatigue resistance, enabling the production of high‐performance components for biomedical and aerospace applications. **Figure** **2** illustrates a schematic of SLM and various commercial implants produced through SLM.

Tensile specimens were fabricated by systematically varying ten distinct parameter sets, each influencing the VED, calculated using Equation (1) as below:

(1) $$VED=\frac{P}{v\cdot h\cdot t}$$

where *P* represents laser power (W), *v* is the scan speed (mm/s), h is hatch spacing (mm), and t is layer thickness (mm). The variations in VED allowed for a detailed study of their effects on mechanical properties such as ultimate tensile strength, yield strength, elongation, and Young's modulus. **Table** **2** states all the processing parameters utilized in the current study for the sample manufacturing.

**Table 2:** Summary of SLM process parameters and corresponding Volumetric Energy Density (VED) values used for fabricating Ti6Al4V specimens.

Sample	Power [W]	Scan Speed [mm/s]	Hatch Distance [mm]	Layer Thickness [mm]	VED
1	200	130	0.12	0.04	321
2	120	125	0.12	0.04	200
3	100	139	0.12	0.04	150
4	350	729	0.12	0.04	100
5	200	555	0.12	0.04	75
6	215	746	0.12	0.04	60
7	280	1167	0.12	0.04	50
8	260	1354	0.12	0.04	40
9	200	1389	0.12	0.04	30
10	200	2083	0.12	0.04	20

### 2.2 Lattice Dataset From Literature

Although focused on dense specimens, extensive literature highlighted SLM's remarkable potential for lattice manufacturing. McGregor et al.^[^ ^31^ ^]^ provided a comprehensive review of lattice designs fabricated using SLM and their mechanical properties, emphasizing their applications in biomedical implants for bone replacement. Lattice structures offer the advantage of tunable porosity and mechanical properties, enabling designs that mimic the hierarchical architecture of trabecular bone. **Table** **3** summarizes examples of lattice structures fabricated using Ti64 powder, along with their mechanical properties such as Young's modulus (E), compressive strength (σ_c_), and yield strength (σ_y_).^[^ ^31^ ^]^

**Table 3:** Comprehensive summary of lattice structures fabricated using SLM, including their porosity, feature thickness, mechanical properties (Young's modulus, compressive strength, and yield strength), and references. This table highlights the variability in lattice designs and mechanical performance, demonstrating the adaptability of SLM for applications such as biomedical implants and lightweight structures.^[^ ^31^ ^]^

Lattice Type	Porosity [%]	Feature thickness [µm]	Young's modulus [GPa]	Compressive strength [MPa]	Yield strength [MPa]	Refs.
Simple cubic	70	400	8.22	168.2	‐	[32]
Diamond	61.6	220	0.66	50	‐	[33]
Rhombic dodecahedron	48.4	300	47.6	422	‐	[34]
Tetrahedron	50	500	4.3	219	‐	[35]
Gyroid TPMS	80	1600	1.25	81.3	‐	[36]
Diamond TPMS	85	500	0.57	65	‐	[37]
Hexagonal honeycomb	67.1	285	3.79	163	110.9	[38]
Stochastic	87.3	210	1.7	550	‐	[39]
Voronoi	88	120	14.3	77.7	1.6	[40]
Octahedron	63	500	5.51	453	228.4	[41]
Triangular honeycomb	50	200	4.3	228	145	[35]
Custom	74.3	600	5.14	195	79.5	[42]
BCC	56.3	600	4.8	333	184.4	[43]
Fluorite	50	1317	3.8	200	200	[44]
Gyroid TPMS	72.6	550	5.58	55.7	34.6	[45]
Porous foam	69	233	‐	‐	‐	[46]
Dodecahedron	92	200	1.6	29.3	22	[47]
Rectangular honeycomb	50	200	‐	‐	‐	[48]
Trabecular	72.6	550	5.58	55.7	34.6	[45]

### 2.3 Mechanical Testing

**Figure 3:**
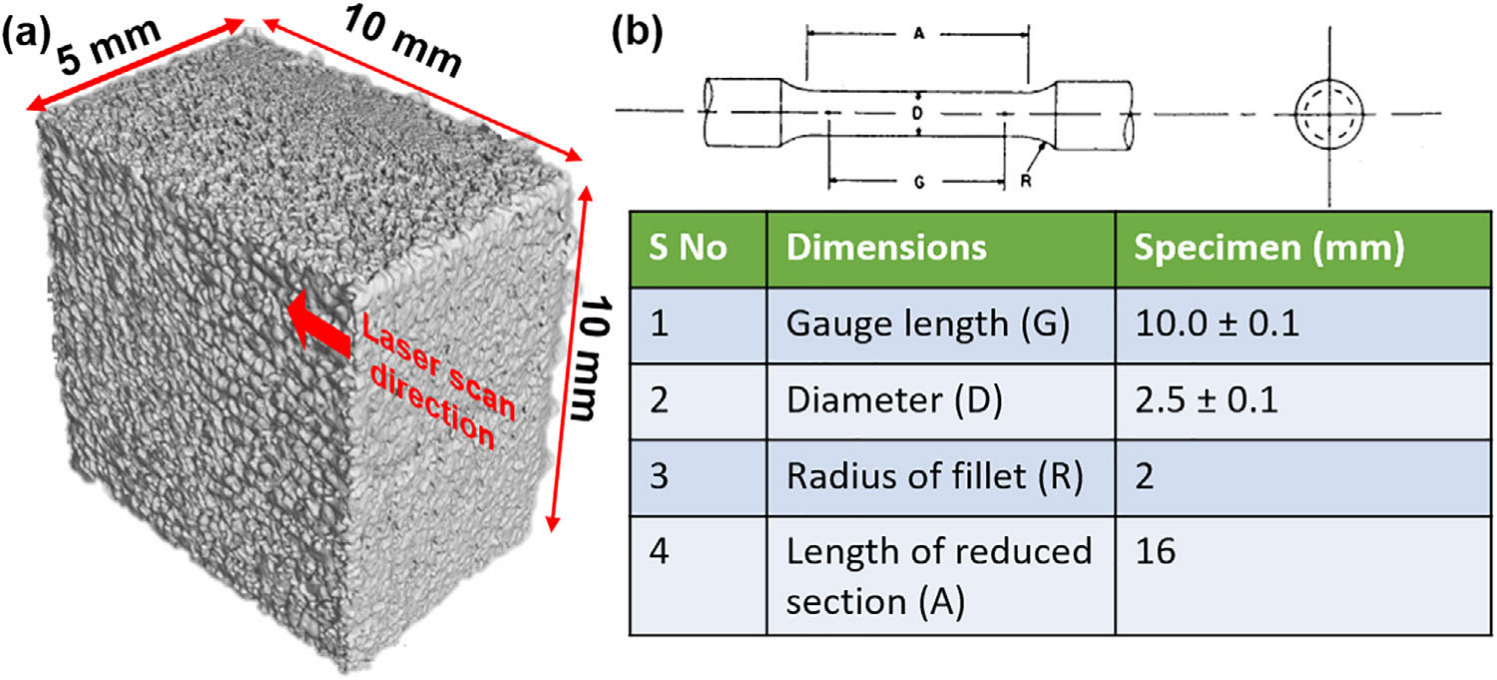
Representation of the fabricated specimens used in this study. a) High‐resolution micro‐computed tomography (micro‐CT) reconstruction of a cubic Ti64 sample (10 × 10 × 5 mm^3^), highlighting its surface topology and structural features. b) Schematic diagram of the ASTM E8 standard tensile specimen with key dimensions and a tabulated summary of its geometrical parameters.

Tensile specimens were fractured with the universal testing machine (Instron 68TM‐50, 50 kN load cell), utilized to evaluate the mechanical properties of the fabricated specimens with a constant crosshead displacement rate of 1 mm min^−1^. The specimen had a gauge length of 10.00 mm and a diameter of 2.50 mm, ensuring standardized testing conditions for reliable mechanical property assessment. Additionally, cubic samples with dimensions of 10 × 10 × 5 mm were fabricated to examine internal porosity and structural integrity through a high‐resolution micro‐computed tomography (micro‐CT) system (Zeiss Xradia 510, Carl Zeiss, Germany). This non‐destructive imaging technique facilitated a detailed analysis of porosity distribution, defect morphology, and volumetric density, providing critical insights into the influence of SLM process parameters on the internal architecture of the fabricated samples. The integration of micro‐CT analysis with mechanical testing establishes a comprehensive framework for correlating process‐induced porosity with mechanical performance, contributing to the optimization of SLM parameters for biomedical applications. **Figure** **3**b presents the schematic and dimensional details of the ASTM E8 tensile specimen, ensuring compliance with standardized mechanical testing requirements. Additionally, Figure 3a shows a high‐resolution micro‐computed tomography (micro‐CT) reconstruction of the cubic sample (10 × 10 × 5 mm^3^), highlighting its surface morphology. The tensile specimens facilitated mechanical property evaluation, while the cubic samples enabled a detailed assessment of process‐induced porosity, contributing to a comprehensive understanding of the influence of SLM parameters on Ti64 structures, which is discussed in detail in the next section. To obtain material properties for finite element simulations, tensile tests were conducted on SLM‐fabricated Ti6Al4V samples produced using varying process parameters (e.g., laser power, scan speed, hatch spacing). All tests were performed according to ASTM E8 standards, and the resulting elastic modulus, yield strength, and ultimate tensile strength values were used as direct inputs in the simulation framework. For lattice configurations not experimentally tested, mechanical properties were obtained from peer‐reviewed literature and verified to fall within the range observed in experiments. Both the tensile specimens and the square implant samples were fabricated using identical SLM parameters, including laser power, scan speed, hatch spacing, and layer thickness, as listed in Table 2. These parameters were systematically varied across builds to study their influence on mechanical performance. The resulting tensile properties—such as elastic modulus and ultimate tensile strength—are presented in Figure 9 and are plotted as a function of VED, allowing direct correlation between processing conditions and material behavior.

### 2.4 Computational Model Development and Calibration

**Figure 4:**
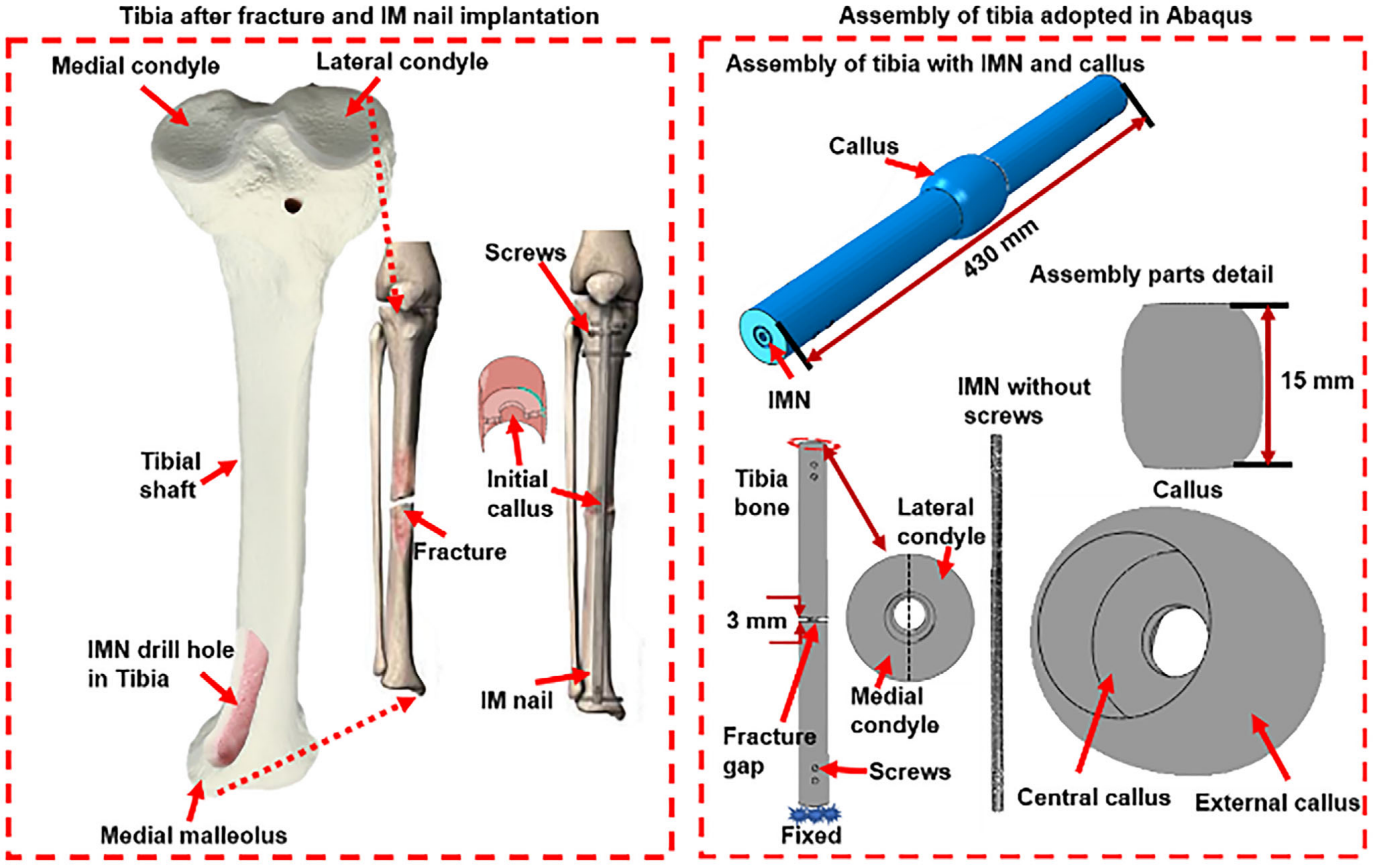
Anatomical structure of the tibia with a mid‐diaphyseal transverse fracture (left) and the finite element model developed in Abaqus CAE for simulating fracture healing (right). The anatomical representation highlights critical bone regions influencing load distribution and callus formation. The computational model includes the tibial shaft, surrounding callus tissue, and an intramedullary (IM) nail with screws for stabilization.

A finite element (FE) model of a human tibia with a 3 mm transverse fracture was developed using SIMULIA Abaqus CAE to simulate the progression of fracture healing under intramedullary nailing (IMN) fixation. The tibia geometry was reconstructed based on anatomical data, with the fracture gap modeled as a 3 mm transverse defect in the diaphyseal region. Initially, the model was created with a small external callus (near‐zero size) to represent the immediate post‐fracture state before callus formation began. A healthy male tibia model featured an outer diameter of 25 mm and an inner diameter of 15 mm to reflect the cortical and medullary regions, respectively. The IMN was simulated as a hollow cylinder with an outer diameter of 8 mm and an inner diameter of 4 mm. To replicate the surgical reaming process, a 1 mm gap was incorporated between the IMN and the inner surface of the bone. A detailed overview of the material properties and meshing information used in the simulation is provided in **Table** **4**. Bone tissue data were sourced from established literature,^[^ ^49^ ^]^ whereas implant properties represent values attainable through additive manufacturing of Ti64, accounting for both solid and lattice configurations. **Figure** **4** illustrates both the anatomical structure of the tibia and the computational model developed for simulating tibial fracture healing in Abaqus CAE. On the left side, the anatomical representation of the tibia is shown, highlighting critical structural features such as the proximal epiphysis (tibial plateau), the diaphyseal shaft, and the distal epiphysis. The tibial shaft, which bears the majority of mechanical loads during daily activities, is emphasized. A transverse mid‐diaphyseal fracture is depicted, which is common in clinical scenarios and represents a challenging fracture type due to its potential for instability. The magnified inset focuses on the fracture site, showcasing the cortical bone, responsible for the bone's strength, and the cancellous bone, essential for biological healing. This part of the figure highlights regions involved in periosteal and endosteal callus formation, crucial for bridging the fracture gap during healing. The anatomical details provide context for understanding how mechanical forces influence different bone regions during the healing process.

**Table 4:** Material properties and meshing parameters used in the finite element analysis. Bone tissue properties (callus, cortical bone, and trabecular bone) were adapted from literature sources.^[^ ^49^, ^50^ ^]^ Mechanical properties for the screws and intramedullary nails were assigned or calculated based on elastic modulus and Poisson's ratio, with IM nail properties representing a range of possible outcomes achievable through additive manufacturing of Ti64, including both solid and lattice structures.

Material	Elastic Modulus [MPa]	Poisson's Ratio	Shear Modulus [MPa]	Porosity	Solid Bulk Modulus [MPa]	Fluid Bulk Modulus [MPa]	Permeability [mm^4^/(N.s)]	Elements Type
Callus‐Granulation Tissues (GTs)	0.2	0.17	0.085	0.8	2300	2300	0.01	C3D8RP
Callus‐Fibrous Tissues (FTs)	5	0.17	2.14	0.8	2300	2300	0.01	
Callus‐Cartilage Tissues (CTs)	500	0.17	21.4	0.8	2300	2300	0.005	
Callus‐Bone Tissues (BTs)	1000	0.3	385	0.8	13920	2300	0.1	
Cortical Bone	Er = 8500, Eθ = 6900, Ez = 18400	νrθ = 0.141, νrz = 0.065, νθz = 0.099	Grθ = 2400, Grz = 4900, Gθz = 3600	0.04	13920	2300	0.00001	C3D8RP
Trabecular Bone	1100	0.26	436.5	0.8	13920	2300	0.1	C3D8RP
Screws (each)	200000	0.3	74230	–	–	–	–	C3D8R
IM Nail	100000	0.3	38461	–	83333	–	–	
	90000	0.3	34615	–	75000	–	–	
	80000	0.3	30769	–	66666	–	–	
	70000	0.3	26923	–	58333	–	–	
	60000	0.3	23076	–	50000	–	–	
	50000	0.3	19230.77	–	41666.67	–	–	
	40000	0.3	15384.62	–	33333.33	–	–	
	30000	0.3	11538.4	–	25000.0	–	–	
	20000	0.3	7692.31	–	16666.67	–	–	
	10000	0.3	3846.15	–	8333.33	–	–	

On the right side, Figure 4 presents the finite element model developed in Abaqus CAE for simulating tibial fracture healing. The top section displays the assembly of the simulation model, which includes a simplified tibial shaft, the callus tissue, and an intramedullary (IM) nail for fracture stabilization. The IM nail is positioned within the medullary canal of the tibia to maintain alignment and support load‐sharing between the bone and the fixation device during healing. The lower section provides a detailed breakdown of the assembly components. The tibial shaft is modeled with precise geometrical features to replicate the structural characteristics of cortical and cancellous bone. The callus is represented as a distinct region surrounding the fracture site, allowing for simulation of its mechanical evolution during healing. The IM nail is modeled with key features such as locking screws and contact interactions with the bone to ensure realistic load transfer and constraint conditions.

**Figure 5:**
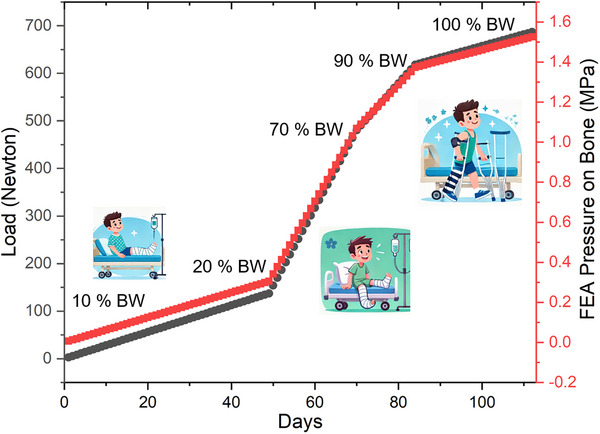
Loading progression during tibial fracture healing simulation. Starting at 10% body weight (BW) for early healing, the load was increased to 70% BW mid‐stage, reaching 100% BW by 16 weeks. Full weight‐bearing (100% BW) represents complete recovery. Images illustrate functional milestones at each stage.

The progression of load bearing following a tibial fracture is critical in optimizing healing outcomes. In the early post‐fracture stage, from zero to seven weeks, partial weight‐bearing is recommended, typically limited to around 10%–20% of the patient's body weight. This initial mechanical stimulation facilitated early callus formation without imposing excessive strain on the fracture site. Sarmiento et al. observed that patients in this phase could tolerate ≈15 kg of axial load, corresponding to around 10% of body weight, without adverse effects. This minimal loading induced interfragmentary movements in the 1–4 mm range, which were beneficial in promoting callus development and subsequent consolidation of the fracture site.^[^ ^51^ ^]^ As the healing process advances into the mid‐stage, between 7 and 16 weeks post‐fracture, there is a significant increase in permissible load bearing. During this phase, the dynamization of external fixators allowed for progressive axial loading, with patients typically bearing 50%–70% of their body weight. This controlled was increased in mechanical load fosters further mineralization and maturation of the callus. Vijayakumar et al. demonstrated that during this mid‐stage, load‐sharing by the external fixator decreased markedly from 20% to 4%, indicating that the healing bone was progressively assuming a greater proportion of the axial load. This shift in load transmission was instrumental in accelerating the healing process while mitigating the risk of overloading the callus, which could otherwise impede recovery or cause refracture.^[^ ^52^ ^]^ By the late stage of healing, typically from sixteen weeks onward, patients can generally bear 90%–100% of their body weight. At this point, the fracture site exhibits substantial stiffness, often exceeding 15 Nm deg^−1^, reflecting the callus's structural integrity and the bone's capacity to support full physiological loads. Joslin et al. reported that patients with well‐healed tibial fractures could achieve weight‐bearing levels approaching 90% of the uninjured leg before the removal of external fixation devices. This strong correlation between increased weight‐bearing capacity and fracture stiffness underscores the effectiveness of using weight‐bearing as a clinical indicator of fracture healing. The gradual progression from minimal to full load‐bearing not only aligns with the biomechanical principles of fracture healing but also minimizes the risk of complications such as delayed union or non‐union, thereby ensuring a robust and efficient recovery process.^[^ ^53^ ^]^ Based on the insights from the literature, a progressive loading protocol was implemented to simulate post‐operative weight‐bearing on the tibia over 16 weeks for a representative 70 kg individual (total body weight force ≈ 686.7 N). The loading was gradually increased from 0% to 100% of body weight in four phases: from 0% to 20% over the first 7 weeks (49 days) with a daily increment of ≈2.80 N; from 20% to 70% over weeks 8–10 (days 50–70) with 13.56 N/day; from 70% to 90% over weeks 11–12 (days 71–84) with 9.80 N/day; and from 90% to 100% during weeks 13–16 (days 85–112) with 2.45 N day^−1^. The corresponding pressure on the tibia was estimated using a cross‐sectional area of 450 mm^2^, resulting in a stress range from 0 to ≈1.53 MPa over the course of the protocol, reflecting a physiologically relevant and controlled increase in mechanical stimulation for bone healing assessment. The outcomes from these load increments were systematically integrated into the current study to evaluate their influence on fracture healing dynamics. The loading pattern is given in **Figure** **5**.

**Figure 6:**
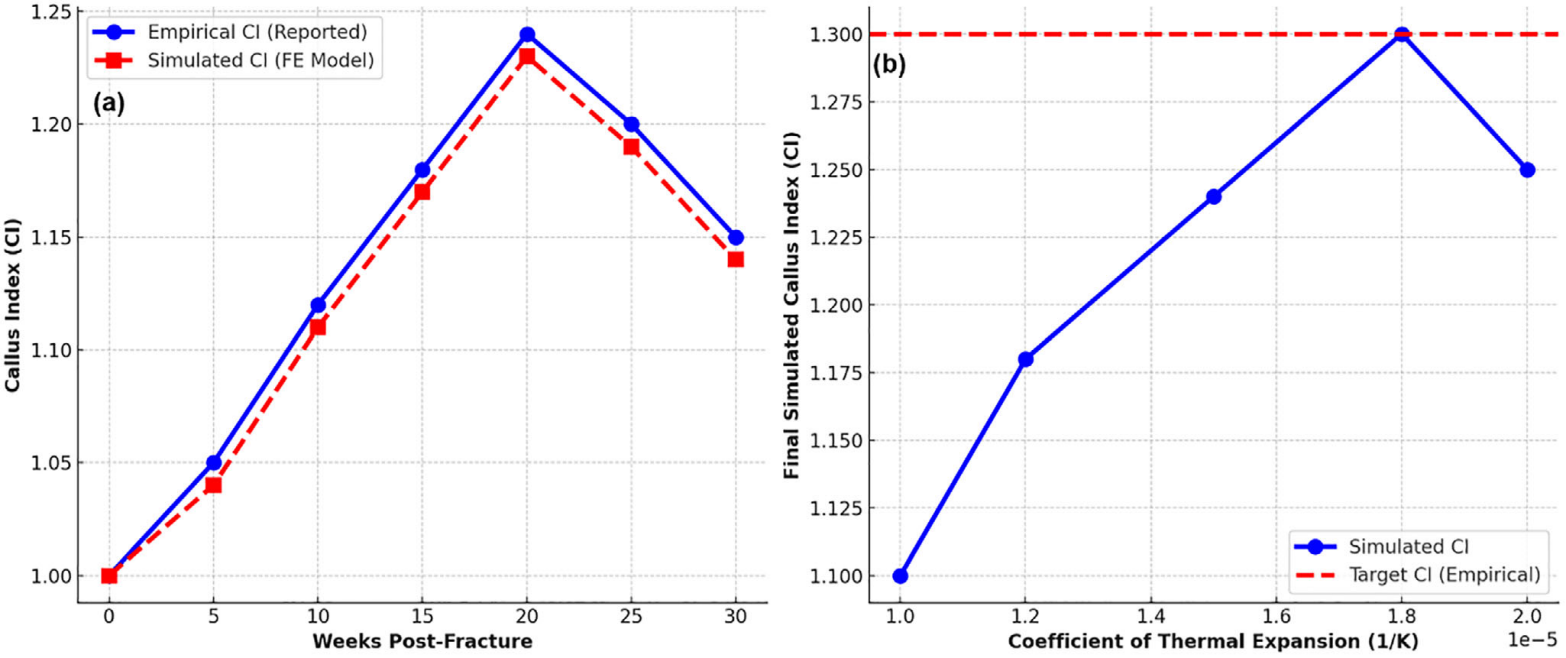
a) Comparison of the empirical Callus Index values reported by Eastaugh–Waring et al.^[^ ^54^ ^]^ With the simulated CI values obtained from the finite element (FE) model. The close agreement between the two datasets validates the model's accuracy in replicating callus growth during fracture healing under intramedullary nail fixation. b) The calibration curve shows the relationship between the CTE and the final simulated CI. The optimal CTE value of 1.8 × 10^−5^ K^−1^ was identified, ensuring that the simulated CI aligns with the peak empirical CI of 1.30. This confirms that the CTE‐based thermal expansion approach effectively models time‐dependent callus growth in the FE simulation.

To ensure the numerical accuracy of the simulation, the callus model expansion was calibrated using the Callus Index (CI) values reported by Eastaugh–Waring et al.^[^ ^54^ ^]^ which quantifies callus growth over time in IMN‐treated tibial fractures. The CI, the ratio of the maximum callus diameter to the bone diameter at the same level, was used to establish a baseline for expected volumetric callus expansion. The simulation was designed to replicate the temporal evolution of the CI, ensuring that the peak CI observed at ≈30 weeks post‐injury in IMN‐treated fractures was accurately reproduced as given in **Figure** **6**a. To ensure that the finite element (FE) model accurately replicates callus growth, a coefficient of thermal expansion (CTE) approach was implemented as a surrogate for biological tissue expansion. In standard thermal expansion, material deformation is governed by Equation (2) as below:

(2) $$\Delta V=V_{o}\cdot \alpha \cdot \Delta T$$

where *α* is the CTE [K−], ΔV represents the change in volume due to expansion, V_0_ is the initial callus volume, and ΔT is the temperature change applied in the simulation. However, in this study, no actual temperature change was applied; instead, a unit temperature change (ΔT = 1) was assumed. This simplification ensured that callus expansion was controlled entirely by the assigned CTE value, allowing the FE solver to interpret the thermal expansion as a growth factor for callus growth. The calibration graph in Figure 6b illustrates the effect of varying CTE values on the final CI achieved in the simulation. The results show a direct correlation between CTE and callus expansion, where higher CTE values resulted in greater volumetric growth. By systematically adjusting the CTE, an optimal value of 1.8 × 10^−5^ K^−1^ was identified, at which the final simulated CI reached 1.30, precisely matching the empirical peak CI reported by Eastaugh–Waring et al.,^[^ ^57^ ^]^ for IMN‐treated tibial fractures. This agreement validates the effectiveness of the thermal expansion analogy as a reliable method for modeling progressive biological growth within the FE framework.

While the CTE‐based approach effectively controlled total callus volume, the shape of the callus was determined by biomechanical stimulus (BMS)‐regulated growth.^[^ ^49^, ^55^ ^]^ This approach dynamically adjusted callus morphology based on local mechanical loading conditions. The effective BMS (ψ_*bms*_) as given in **Table** **5** was defined to account for both shear deformation and fluid‐induced mechanotransduction calculated according to Equation (3), as follows:

(3) $$\psi_{bms}\left(x,t\right)=\beta_{1}\frac{\varepsilon_{d}}{\varepsilon_{ref}}+\beta_{2}\frac{v_{f}}{v_{ref}}+\beta_{3}\frac{p}{p_{ref}}$$

where

- *ε_d_* represents the deviatoric strain, which influences tissue differentiation. Higher strain promotes fibrous tissue formation, while moderate strain supports bone growth.^[^ ^55^ ^]^
- *ν_f_* is the fluid velocity, which plays a crucial role in cartilage development via fluid‐driven mechanotransduction.^[^ ^55^ ^]^
- *p* is the local pore pressure, which plays a key role in tissue differentiation.^[^ ^50^ ^]^
- β_1_, β_2,_ and β_3_ are threshold values that define tissue response boundaries. β1 = 3.75, β2 = 3.0, and β_3_ = 2.5, ensuring scaling factors remain consistent with the literature.^[^ ^50^ ^]^

**Table 5:** Ψ_bms_ range according to the tissue formation.

Tissue type	Biomechanical stimulus threshold (ψ(x,t))^[^ ^55^, ^56^ ^]^
Fibrous tissue formation	Ψ_bms_(x,t)>3.0
Cartilage formation	1.0<Ψ_bms_(x,t)≤3.0
Bone formation	0.01≤Ψ(x,t)_bms_≤1.0

This equation ensures that callus formation is optimized based on local mechanobiological conditions, where higher BMS values lead to increased tissue deposition, and low BMS values cause tissue resorption.^[^ ^55^, ^57^ ^]^ The Abaqus subroutine continuously updates ψ_bms_, dynamically modifying the callus geometry over time.

To incorporate implant stiffness effects, the growth scaling coefficient (β) was formulated to ensure that fixation rigidity influenced callus expansion patterns calculated according to Equation (4) as below:

(4) $$\beta =\lambda \left(1-\frac{E_{implant}}{E_{bone}}\right)$$

where

- λ is a scaling factor that adjusts how much the difference between implant stiffness (E_implant_) and bone stiffness (E_bone_) affects the final value of β.
- Lower E_implant_ (more flexible implant) → greater callus growth due to increased micromotion‐induced mechanostimulation.
- Higher E_implant_ (stiffer implant) → limited callus formation, leading to more direct bone bridging instead of an extended external callus.

Since *β* controls how much biomechanical stimulus affects growth, it modifies the ψ_bms_ term in the volume growth equation, which is then calculated by Equation (5) as follows:

(5) $$\psi_{avg}=\beta .\psi_{bms}$$

Thus, the final equation for callus volume change, incorporating all mechanical and biological factors, is given as Equat (6) below:

(6) $$\frac{\Delta V}{V}=\xi \cdot \beta \cdot \psi_{bms}\cdot \left(\frac{\rho_{msc}}{\rho_{max}}\right)\cdot \frac{\partial \rho_{msc}}{\partial t}$$

where

- $\frac{\Delta V}{V}$ Represents the relative volume increase of the callus over time.
- ξ is a growth rate coefficient, ensuring appropriate calibration with empirical data.
- ψ_bms_ is the biomechanical stimulus at a given time and position.
- ρ_msc_ is the local mesenchymal stem cell (MSC) concentration, which governs osteogenic activity.
- ρ_max_ represents the maximum stem cell concentration, ensuring the model respects biological limits.
- $\frac{\partial_{\rho_{msc}}}{\partial t}$ The rate of MSC proliferation modulates how quickly new tissue is formed.

This equation ensures that callus volume changes are influenced not only by mechanical stimulus but also by the biological availability of precursor cells, leading to more physiologically accurate healing predictions

### 2.5 Finite Element Approach for Callus Evolution and Bone Healing

**Figure 7:**
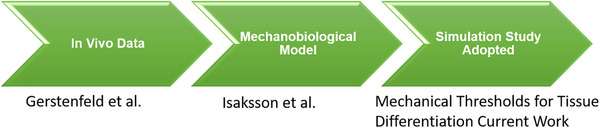
Schematic representation of the model validation pathway. The mechanobiological model used in this study was originally calibrated against in vivo data (Gerstenfeld et al.)^[^ ^60^ ^]^ and further developed by Isaksson et al.^[^ ^49^, ^50^ ^]^ The present simulation framework adopts the same mechanoregulation principles and parameter thresholds, ensuring biological relevance in predicting bone healing around SLM‐Ti64 implants.

The bone healing simulation in Abaqus was developed using a Python‐based subroutine, allowing for real‐time updates of tissue properties based on mechanical loading, cell migration, tissue differentiation, and remodeling. This model builds upon the Callus Growth Model (CGM), where callus formation was influenced not only by CTE but also by implant stiffness and diffusion‐driven cell movement. These factors collectively controlled the spatial and temporal progression of healing, ensuring that callus formation was not uniform but regulated by mechanical and biological stimuli.^[^ ^55^, ^58^ ^]^ The mechanobiological model employed in this study was calibrated based on the framework developed by Isaksson et al.^[^ ^59^ ^]^ and Gerstenfeld et al.^[^ ^60^ ^]^ and further refined in Isaksson et al.^[^ ^49^ ^]^ These models were validated against in vivo histological and radiographic data from healing ovine tibial fractures, under controlled loading conditions. The same thresholds for deviatoric strain and fluid flow used to guide tissue differentiation in those studies were adopted here to ensure biological consistency. The calibration flow is depicted in **Figure** **7**.

At the early stage of healing, mesenchymal stem cells (MSCs) migrate into the fracture site, responding to chemical gradients and mechanical forces. The movement of MSCs was modeled using a diffusion‐reaction Equation (7), ensuring that their distribution followed random motion and directed migration toward areas with optimal healing conditions^[^ ^49^ ^]^ as below:

(7) $$\frac{\partial C_{MSC}}{\partial t}=D\nabla^{2}C_{MSC}-\nabla \cdot \left(\mu_{MSC}\nabla \psi_{bms}\left(x,t\right)\right)$$

where

- *C_MSC_* is the MSC concentration at time t,
- *D* is the diffusion coefficient, governing random movement,
- *ψ_bms_(x,t)* represents the mechanoregulation stimulus, as defined in CGM,
- *µ_MSC_* ​is the directed migration coefficient, ensuring MSCs move toward areas of optimal mechanical conditions.

The concentration of each cell type evolves according to Equation (8):

(8) $$\frac{\partial c_{i}}{\partial t}=M_{i}+P_{i}+F_{i}-A_{i}$$

where

- *c_i_* is the concentration of a specific cell type (MSC, fibroblast, chondroblast, osteoblast),
- *M_i_* is the migration rate,
- *P_i_* is the proliferation rate,
- *F_i_* is the differentiation rate,
- *A_i_* is the apoptosis rate.

The biological processes for each cell phenotype were modeled as in **Table** **6**.

**Table 6:** Cell phenotype‐related activities adopted in the simulation process.

Process	Equation	Parameter Choice & Justification
Migration (*M_i_*)	M_i_ = ▽.(D_i_▽c_i_)	∇*D_i_* based on experimental MSC diffusion rates^[^ ^61^ ^]^
Proliferation (*P_i_*)	*P_i_ = k_Pi_·c_i_·(1‐ c_i_ ·(1+λψ(x,t))*	*k_Pi_*, λ derived from MSC proliferation rates
Differentiation (*F_i_*)	$F_{i}=\sum_{m=1}^{4,m\neq 1}\left(k_{F_{m,i}}.c_{m}\right)$	Differentiation follows established cell lineage transitions
Apoptosis (*A_i_*)	*A_i_ = k_Ai._c_i_*	*k_Ai_* matching literature‐based apoptosis rates

The term *k_Pi_* is the proliferation rate constant, which quantifies how fast a specific cell type divides and expands in a given period. It is expressed in day^−1^, meaning it represents the fraction of cells that undergo division per day. The term *k_Ai_* is the apoptosis rate constant, which quantifies how fast a specific cell type undergoes programmed cell death. Like *k_Pi_*, it is expressed in day^−1^, meaning it represents the fraction of cells that die per day. The parameter *k_Fm,i_* represents the differentiation rate constant for the transition of one cell phenotype (*m*) into another (*i*) during bone healing. Unlike proliferation (*k_Pi_*), which controls how fast cells divide, and apoptosis (*k_Ai_*), which governs cell death, differentiation rate constants define how quickly one type of cell transforms into another, contributing to the overall tissue maturation process. Following in **Table** **7** represents the proliferation and apoptosis rate as defined in the literature and adopted in the current simulation process.^[^ ^49^, ^57^ ^]^

**Table 7:** Cell proliferation and apoptosis rate adopted in the simulation process.

Cell Type	Proliferation rate (*k_Pi_*) [day^−1^]	Apoptosis rate (*k_Ai_*) [day^−1^]
MSC	0.6	0.05
Fibroblast	0.55	0.05
Chondrocyte	0.2	0.1
Osteoblast	0.3	0.15

As healing progressed, cartilage underwent endochondral ossification, transforming into mineralized woven bone.^[^ ^61^ ^]^ This phase was modeled using a time‐dependent tissue stiffness update, ensuring that as ψ(x,t) evolved^[^ ^62^ ^]^ as given in Equation (9), soft tissues stiffened into bone, while its details are given in **Table** **8**:

(9) $$E_{tissue}\left(t\right)=E_{o}+\gamma \cdot \int_{to}^{t}(\psi \left(x,t\right)-\psi_{threshold}H\left(\psi \left(x,t\right)-\psi_{threshold}\right)d_{t}$$

**Table 8:** Explanation matrix for Equation (9).

Symbol	Meaning	Explanation
*E_tissue_(t)*	Tissue stiffness at time (t)	The stiffness (elastic modulus) of the healing tissue at a given time (t). This value increases as tissue mineralizes over time.
*E_0_*	Initial tissue stiffness	Represents the starting stiffness of the healing tissue. Initially, soft callus has a lower stiffness, which later increases with mineralization.
*γ*	Growth rate coefficient	Determines how quickly stiffness evolves based on biomechanical stimulus.
$\int_{to}^{t}\left(\right)$	Integral over time	Represents the accumulation of stiffness changes over time. This ensures that past mechanoregulation effects contribute to future stiffness evolution.
*Ψ(x,t)*	Biomechanical stimulus	The mechanical environment at a given location (x) and time (t).
*ψ_threshold_*	Threshold stimulus value	Defines the minimum mechanical stimulus required for bone mineralization.
*H(ψ(x,t)‐ ψ_threshold_)*	Heaviside function	Ensures that stiffness only increases when *Ψ(x,t) > ψ_threshold_*. If *Ψ(x,t) < ψ_threshold_*, the function becomes 0, preventing stiffness evolution.

Once mineralization was complete, the final remodeling phase began, where osteoclasts resorbed woven bone and replaced it with lamellar bone, optimizing its mechanical strength and structure. This process was modeled as Equation (10), while details of the equation are given in **Table** **9**:

(10) $$E_{tissue}^{n+1}=E_{tissue}^{n}+\Delta t\cdot f_{adaptation}\left(\psi \left(x,t\right)\right)$$

**Table 9:** Explanation for the remodeling phase of the simulation, as in Equation (10).

Symbol	Meaning	Explanation
$E_{tissue}^{n+1}$	Tissue stiffness at time (n+1)	The updated stiffness of the healing bone at the next time step after remodeling.
$E_{tissue}^{n}$	Tissue stiffness at time (n)	The current stiffness value before the remodeling update.
▽t	Time step increment	The time interval between two updates in the simulation, typically 1 day in most bone healing models.
*f_adaptation_* (ψ(*x*,*t*))	Bone adaptation function	Describes how bone stiffness evolves in response to biomechanical stimulus *(ψ_bms_(x,t))*.

### 2.6 Summary of the Simulation Process

The full bone healing simulation approach is described in Sections 2.2 and 2.3 of this manuscript. The model used is based on a previously validated mechanobiological framework that predicts how different tissues, such as fibrous tissue, cartilage, and bone, form during healing based on the local mechanical environment. This model is widely used in literature and is built on the principle that mechanical cues like strain and fluid pressure regulate how cells behave during healing. These cues determine whether the tissue in a specific location turns into cartilage, bone, or remains fibrous. At the start of the simulation, the defect region was assumed to be filled with granulation tissue. Over time, the local mechanical conditions, calculated using finite element analysis, guide how this tissue changes. If the mechanical strain and fluid flow are low, the environment is favorable for direct bone formation, known as intramembranous ossification. If the strain is moderate, cartilage is formed first, which is then replaced by bone, a process known as endochondral ossification. If the mechanical conditions were too unstable, such as under high strain or excessive fluid flow, fibrous tissue forms instead, which is less favorable for bone regeneration. These tissue‐specific thresholds were adopted from previously published studies by Isaksson et al.^[^ ^49^, ^50^ ^]^ and Shefelbine et al.,^[^ ^63^ ^]^ who validated them using experimental data from animal models.

Along with the mechanical conditions, biological processes such as cell migration, proliferation, and differentiation were also simulated. The model assumed that cells migrate into the defect area and differentiate into either fibroblasts, chondrocytes, or osteoblasts, depending on the local strain and fluid conditions. As these cells deposit extracellular matrix, the tissue in the defect changes its stiffness and structure, which in turn alters the mechanical environment for the next time step in the simulation. This feedback loop continues over the entire healing period, which was simulated over 112 days in the study.

No new experimental measurements of cellular responses were conducted in this study. Instead, the biological parameters and rate constants used in the simulation, such as how fast cells migrate or how quickly tissue changes occur, were directly taken from the validated literature models. The simulation was designed to predict spatiotemporal healing behavior around implants with different mechanical properties, enabling to assess how variations in implant stiffness affect bone regeneration over time. This approach allows for a biologically meaningful prediction of healing outcomes, based on both mechanical and cellular behavior, and forms the foundation of the machine learning model developed in the latter part of this study. The bone healing simulation models mechanically regulated cellular behavior, integrating biomechanical stimulus, cell migration, differentiation, proliferation, apoptosis, and tissue remodeling. The biomechanical stimulus function (ψ_bms_(x,t)) determines tissue differentiation based on deviatoric strain, fluid velocity, and pore pressure, calculated as:

(11) $$\psi_{bms}\left(x,t\right)=\beta_{1}\frac{\varepsilon_{d}}{\varepsilon_{ref}}+\beta_{2}\frac{v_{f}}{v_{ref}}+\beta_{3}\frac{p}{p_{ref}}$$

where *β1,β2,β3* ​are scaling factors. Tissue differentiation follows:

(12) $$\left\{\begin{array}{c} Bone\;formation,0.01\leq \psi bms\left(x,t\right)\leq 1.0\\ Cartilage\;formation,1.0<\psi bms\left(x,t\right)\leq 3.0\\ Fibrous\;T\;formation,\psi bms\left(x,t\right)>3.0\\ \;Resorption\;\psi bms\left(x,t\right)\leq 0.01 \end{array}\right\}$$

Cell migration follows a diffusion‐reaction model, where cells diffuse randomly and migrate toward high mechanical stimulus:

(13) $$\frac{\partial C_{MSC}}{\partial t}=D\nabla^{2}C_{MSC}-\nabla \cdot \left(\mu_{MSC}\nabla \psi_{bms}\left(x,t\right)\right)$$

The change in cell population over time is governed by:

(14) $$\frac{\partial c_{i}}{\partial t}=M_{i}+P_{i}+F_{i}-A_{i}$$

(15) $$Pi=k_{pi}\cdot c_{i}\cdot \left(1-c_{i}\right)\cdot \left(1+\lambda \psi_{bms}\left(x,t\right)\right)$$

(16) $$F_{i}=\sum_{m=1}^{4,m\neq i}\left(k_{F_{m,i}}\cdot c_{i}\right)$$

(17) $$A_{i}=k_{A_{i}}\cdot c_{i}$$

Tissue stiffness evolves dynamically based on accumulated mechanical stimulus:

(18) $$E_{tissue}\left(t\right)=E_{o}+\gamma \cdot \int_{to}^{t}(\psi \left(x,t\right)-\psi_{threshold}H\left(\psi \left(x,t\right)-\psi_{threshold}\right)d_{t}$$

(19) $$E_{tissue}^{n+1}=E_{tissue}^{n}+\Delta t.f_{adaptation}\left(\psi \left(x,t\right)\right)$$

The simulation process keeps going until 112 days with updated callus properties in an iterative manner. After each iteration amount of bone development is quantified and applied to the next iteration. This computational bone healing simulation, developed using Abaqus and Python‐based subroutines, integrates biomechanical stimulus, cellular behavior, and tissue remodeling principles to provide a biologically accurate model of fracture healing. By incorporating mechanoregulation thresholds, cell migration dynamics, phenotype‐specific proliferation and apoptosis rates, and adaptive tissue stiffness evolution, the model ensures that bone healing progresses in response to mechanical cues. The equations governing biomechanical stimulus ψ(x,t), cell population dynamics, and bone adaptation are validated against experimental literature, ensuring that callus formation, chondrocyte‐driven endochondral ossification, and osteoblast‐mediated mineralization follow physiologically realistic patterns. The stepwise numerical calculations for MSC migration, proliferation, apoptosis, and tissue stiffness changes confirm that the simulation effectively predicts cellular responses and mechanical property evolution at each stage of healing. By dynamically adjusting tissue stiffness and cell behavior based on mechanical loading, this model provides a robust framework for understanding fracture healing under various implant conditions, mechanical environments, and biological stimuli, making it a valuable tool for biomechanics research, implant design, and regenerative medicine applications.^[^ ^49^ ^]^ The mechanical properties used in simulations were obtained from tensile tests on solid SLM‐fabricated Ti6Al4V samples, while lattice structure properties were taken from the literature. Although the mechanobiological model is based on fracture healing simulations, it remains applicable to peri‐implant healing in mechanically stabilized environments where tissue formation is driven by local strain. It is acknowledged that it does not capture surface‐driven osseointegration mechanisms, which will be addressed in future model extensions.

### 2.7 Statistical Analysis

All mechanical testing results are reported as mean ± standard deviation (SD) based on a minimum of three replicates per parameter set (*n* = 3). For ML model evaluation, the dataset was pre‐processed by normalizing input features and removing outliers using the interquartile range (IQR) method. Performance of regression models was assessed using standard statistical metrics, including coefficient of determination (R^2^), root mean square error (RMSE), and mean absolute error (MAE) across both validation and test datasets.

Since this study focuses on simulation‐based predictions and not group‐wise experimental comparisons, no formal hypothesis testing was applied. All analyses were conducted using Python (v3.10), with relevant packages including scikit‐learn, NumPy, Pandas, and FEA using Dassault Systèmes Simulia Corp. (2023). Abaqus 2023. Providence, RI, USA. All data are presented as mean ± standard deviation (*n* = 3) per VED condition. A significance threshold of *p* < 0.05 is used as a standard for all porosity and micro‐CT scans data presented.

## 3 Results and Discussion

### 3.1 Correlative Analysis of Microstructural Defects and Mechanical Performance

The relationship between VED and the microstructural integrity of Ti64 components fabricated via SLM reveals a complex interplay between energy input, porosity formation, and defect morphology. The quantitative analysis of porosity distribution across different VED values provides insights into how processing parameters influence material densification, highlighting an optimal energy range that minimizes defect formation while preserving mechanical strength.^[^ ^64^ ^]^

**Figure 8:**
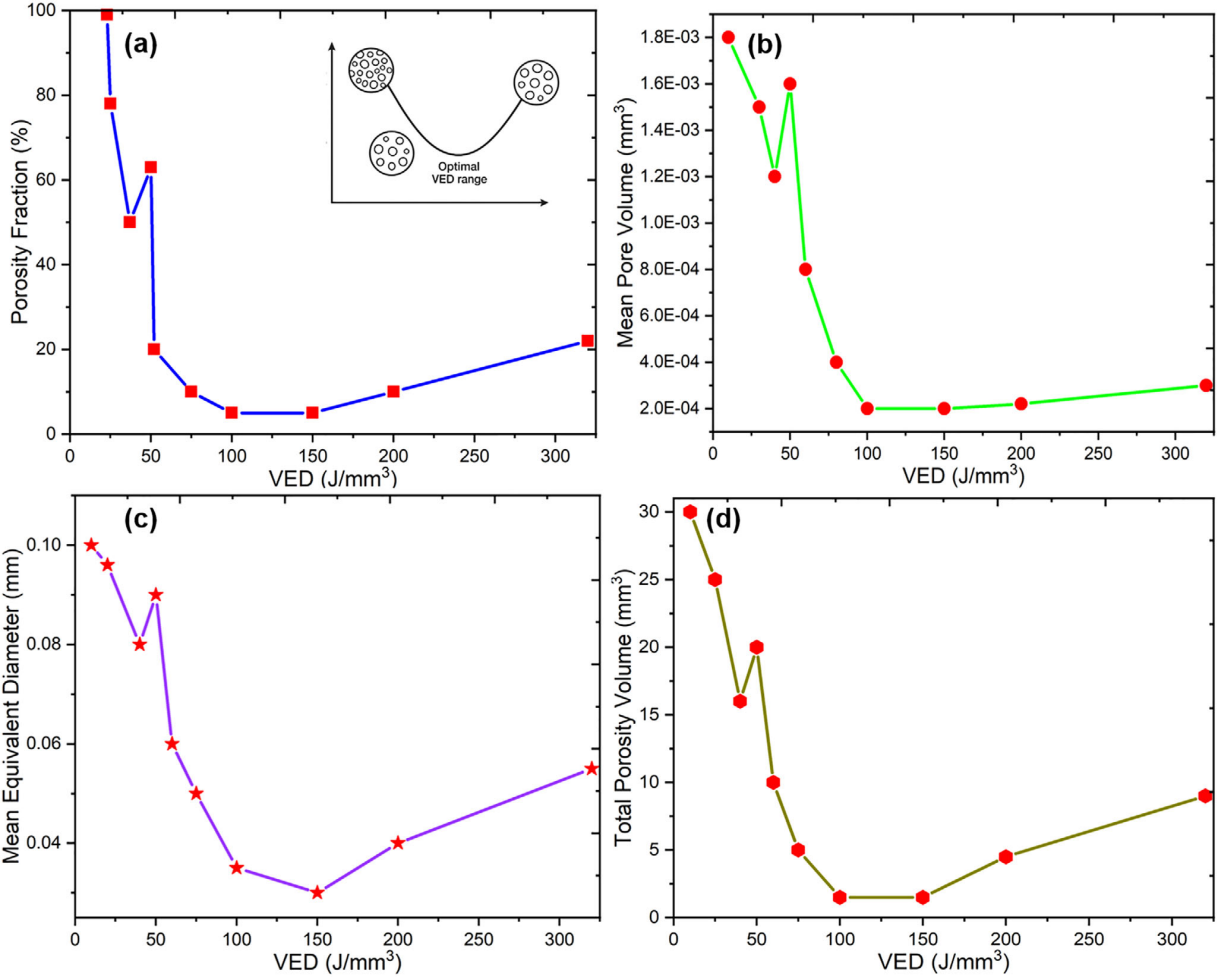
Relationship between Volume Energy Density (VED) and porosity characteristics in SLM‐fabricated Ti64 specimens. a) Mean pore volume as a function of VED, showing a significant reduction in porosity beyond 50 J mm^−^^3^ due to improved melting conditions. b) Mean equivalent pore diameter versus VED, illustrating the transition from lack‐of‐fusion defects at low VED to keyhole porosity at high VED. c) Total porosity volume versus VED, highlighting the optimal energy range (75–150 J mm^−^^3^) for minimal defect formation. d) Porosity fraction as a percentage of total defect volume, reinforcing the densification behavior across different energy levels. All data are presented as mean ± standard deviation (*n* = 3) per VED condition. Statistical comparisons were performed using one‐way ANOVA followed by Tukey's post‐hoc test. ^*^ *p* < 0.05, ^**^ *p* < 0.01, ns: not significant.

A significant transition is observed at 50 J mm^−^^3^ VED, where porosity metrics experience a sudden shift. The mean pore volume is significantly higher at lower VED values, particularly below 50 J mm^−^^3^, indicating poor fusion between powder layers due to insufficient energy input. This phenomenon arises when the energy imparted by the laser is inadequate to fully melt and consolidate the powder particles, leading to lack‐of‐fusion defects. These defects are characterized by irregularly shaped, large voids distributed non‐uniformly throughout the structure.^[^ ^64^ ^]^ However, as the VED increases beyond 50 J mm^−^^3^, a critical threshold is crossed where melting conditions improve significantly, leading to a sharp decline in porosity. This abrupt transition suggests that 50 J mm^−^^3^ is where the melt pool stabilizes, powder absorptivity improves, and material fluidity is enhanced, facilitating more complete fusion. The sudden reduction in porosity indicates that beyond this threshold, the laser energy input becomes sufficient to minimize interlayer voids and promote a more uniform densification process.^[^ ^64^, ^65^ ^]^ This effect is further influenced by scan speed,^[^ ^65^ ^]^ where at 50 J mm^−^^3^ (200 W, 1389 mm s^−1^), the balance between energy input and laser movement allows for improved material consolidation compared to lower VED values with excessively high scan speeds as shown in **Figure** **8**a–d.

As the VED further increases, reaching ≈100–150 J mm^−^^3^, the mean pore volume decreases, suggesting improved densification and reduced porosity. This energy range appears to provide sufficient melting and wetting of the powder particles, facilitating strong interlayer bonding and minimizing the formation of voids.^[^ ^64^ ^]^ However, beyond 200 J mm^−^^3^, a slight increase in mean pore volume is observed, marking the onset of keyhole porosity, a phenomenon commonly associated with excessive laser energy input. In this regime, localized overheating leads to the formation of deep vaporization cavities that collapse and trap gas, generating elongated and unstable voids within the material. The role of low scan speed at high VED (e.g., 321 J mm^−^^3^, 200 W, 130 mm s^−1^) becomes increasingly significant, as prolonged exposure to the laser results in excessive energy absorption, leading to the formation of unstable keyhole defects as illustrated in Figure 8b. The equivalent pore diameter follows a similar trend, further reinforcing the transition between lack‐of‐fusion and keyhole porosity as the VED increases. At low VED values, the equivalent pore diameter is relatively large, reflecting the presence of coarse, irregular defects that result from incomplete melting and weak powder fusion. As the VED increases to the optimal range of 75–150 J mm^−^^3^, the equivalent pore diameter decreases, indicating that defects become finer and more uniformly distributed. This suggests that the melting conditions in this energy range promote better material consolidation, reducing the presence of large interfacial voids. However, at very high VED values exceeding 200 J mm^−^^3^, an increase in equivalent pore diameter is observed again. This implies that keyhole porosity introduces larger, elongated defects compared to the irregular voids seen at lower energy densities.^[^ ^66^ ^]^ This distinction is important, as the formation of keyhole porosity can have different mechanical implications compared to lack‐of‐fusion defects. While lack‐of‐fusion voids often result in weak interlayer bonding and brittle failure, keyhole porosity can act as crack initiation sites due to the sharp stress concentration effects associated with elongated defects, as shown in Figure 8c. The total porosity volume further substantiates the relationship between VED and porosity formation, demonstrating a sharp decline as VED increases from 20 J mm^−^^3^ to ≈100 J mm^−^^3^. This confirms that increasing energy input improves material densification by reducing the prevalence of unfused powder regions. However, beyond 200 J mm^−^^3^, the total porosity volume rises again, signaling the emergence of keyhole‐induced defects. The porosity fraction, which represents the normalized total porosity volume, follows a comparable trend, reinforcing that an optimal VED range exists where the material achieves its highest density while avoiding the detrimental effects of excessive energy input. Additionally, the influence of scan speed becomes apparent, as extremely high speeds at low VED values (e.g., 2083 mm s^−1^ at 20 J mm^−^^3^) exacerbate lack‐of‐fusion defects by reducing the effective energy absorption, while excessively low scan speeds at high VED values prolong heat exposure, leading to keyhole instability as given in Figure 8d. **Table** **10** provides the calculated porosity percentage in the samples over different parameters of SLM.

**Table 10:** Porosity Metrics for SLM‐Fabricated Ti64 samples. Summary of key porosity characteristics, including porosity fraction, total porosity volume, mean pore volume, and mean equivalent pore diameter, as a function of volumetric energy density (VED). The data highlights the transition from lack‐of‐fusion porosity at low VED to optimal densification in the 75–150 J mm^−^^3^ range, followed by keyhole porosity at higher VED values (>200 J mm^−^^3^).

VED [J mm^−^^3^]	Porosity fraction [%]	Total porosity volume [mm^3^]	Mean pore volume (mm^3^)	Mean equivalent diameter [mm]
20	6	30	0.0018	0.1
30	5	25	0.0015	0.095
40	3.6	18	0.0012	0.08
50	4	20	0.0016	0.09
60	2	10	0.0008	0.06
75	1	5	0.0004	0.05
100	0.4	2	0.0002	0.035
150	0.4	2	0.0002	0.03
200	0.8	4	0.00025	0.04
321	1.6	8	0.0003	0.055

**Figure 9:**
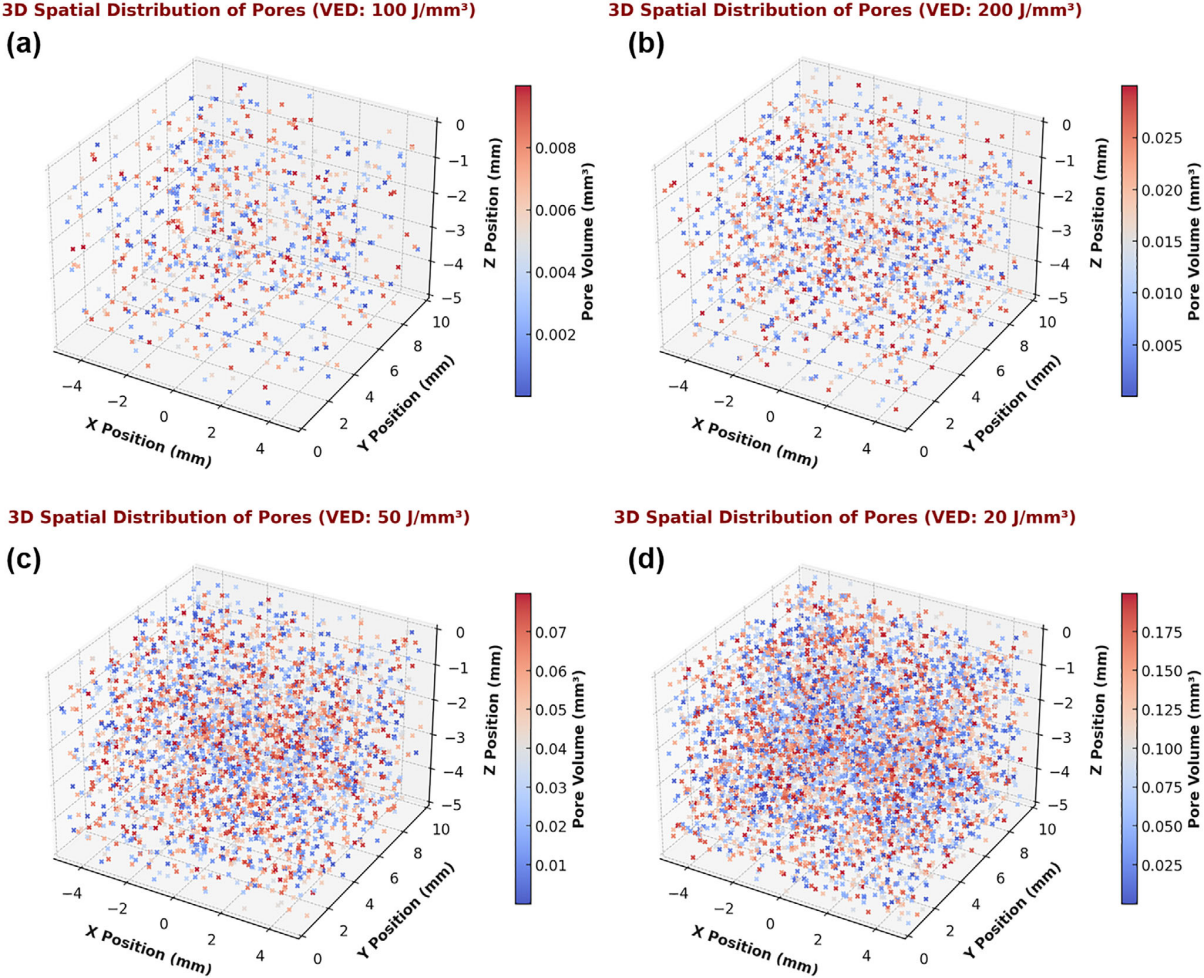
3D spatial distribution of pores in SLM‐fabricated Ti64 specimens at different Volume Energy Densities (VEDs). a) 100 J/mm^3^ demonstrates a uniform distribution of small, well‐dispersed pores, indicating optimal densification. b) 200 J mm^−^^3^ shows the presence of larger voids due to keyhole porosity, caused by excessive laser energy input. c) 20 J mm^−^^3^ exhibits highly dispersed and large‐scale porosity, characteristic of lack‐of‐fusion defects due to insufficient melting. d) 50 J mm^−^^3^ marks the transition phase where porosity reduces as the melt pool stabilizes, but localized void clusters are still evident. The color scale represents pore volume, with red regions indicating larger defects. All data are presented as mean ± standard deviation (*n* = 3) per VED condition. Statistical comparisons were performed using one‐way ANOVA followed by Tukey's post‐hoc test. ^*^ *p* < 0.05, ^**^ *p* < 0.01, ns: not significant.

The spatial distribution of pores provides further insights into the defect formation mechanisms across different energy densities. At low VED (20 J mm^−^^3^), the pore distribution is widespread, with large, irregular voids predominantly caused by lack‐of‐fusion defects due to insufficient energy input. The limited melting results in weak interlayer bonding, leading to extensive porosity that compromises mechanical integrity. As the VED increases to 50 J mm^−^^3^, a noticeable transition occurs, where porosity declines. However, localized clusters of voids persist, indicating that while melt pool stability improves, complete fusion has not yet been achieved. This transitional phase suggests that the laser energy is nearing the threshold required to melt the powder fully, but is still insufficient for complete densification. In contrast, at 100 J mm^−^^3^, the spatial distribution of pores shows a significant refinement, characterized by small, uniformly distributed voids indicative of optimal densification. This energy input ensures thorough melting and fusion of powder particles, reducing overall porosity and leading to a homogeneous microstructure with minimal internal defects. However, as the VED increases to 200 J mm^−^^3^, keyhole porosity becomes evident, marked by larger, elongated voids resulting from excessive laser energy input. The formation of deep vaporization pits at high VED causes gas entrapment, leading to localized defects where the melt pool becomes unstable due to over‐penetration of the laser. These observations highlight the nonlinear influence of VED on porosity formation, demonstrating that lack‐of‐fusion defects at low VED and keyhole porosity at high VED can detrimentally impact material integrity. **Figure** **9**a–d shows the spatial distribution of pores for the comparison at different VED values.

From a biomedical perspective, achieving a controlled porosity distribution is essential to ensure mechanical reliability and biological functionality. In orthopedic and dental implants, Ti64 components must exhibit high strength while maintaining a degree of porosity that facilitates osseointegration. Excessive porosity can compromise mechanical stability, while insufficient porosity may hinder bone integration due to a lack of surface roughness. The results of this study suggest that the optimal VED range of 75–150 J mm^−^^3^ provides the best balance between these competing requirements. The improved densification achieved in this range enhances structural integrity while maintaining a microstructure conducive to biological interactions.

The impact of these porosity characteristics on mechanical performance is substantial, as the formation of internal voids directly affects the strength, fatigue life, and fracture resistance of Ti64 components. High porosity levels at low VED values significantly reduce mechanical strength, as these defects act as stress concentrators that facilitate crack initiation and propagation. Large, irregular voids weaken interlayer bonding, making the material susceptible to premature failure under mechanical loading. In contrast, the optimal VED range (75–150 J mm^−^^3^) results in minimal porosity, translating to superior tensile strength and fatigue resistance. The more minor, more evenly distributed defects observed in this range contribute to enhanced load‐bearing capacity, as stress is distributed more uniformly throughout the material. However, at excessive VED values, despite achieving lower overall porosity compared to low‐energy samples, the presence of keyhole porosity introduces new failure mechanisms. These elongated defects can lead to localized stress concentrations, significantly affecting fatigue performance by promoting crack growth under cyclic loading conditions.

**Figure 10:**
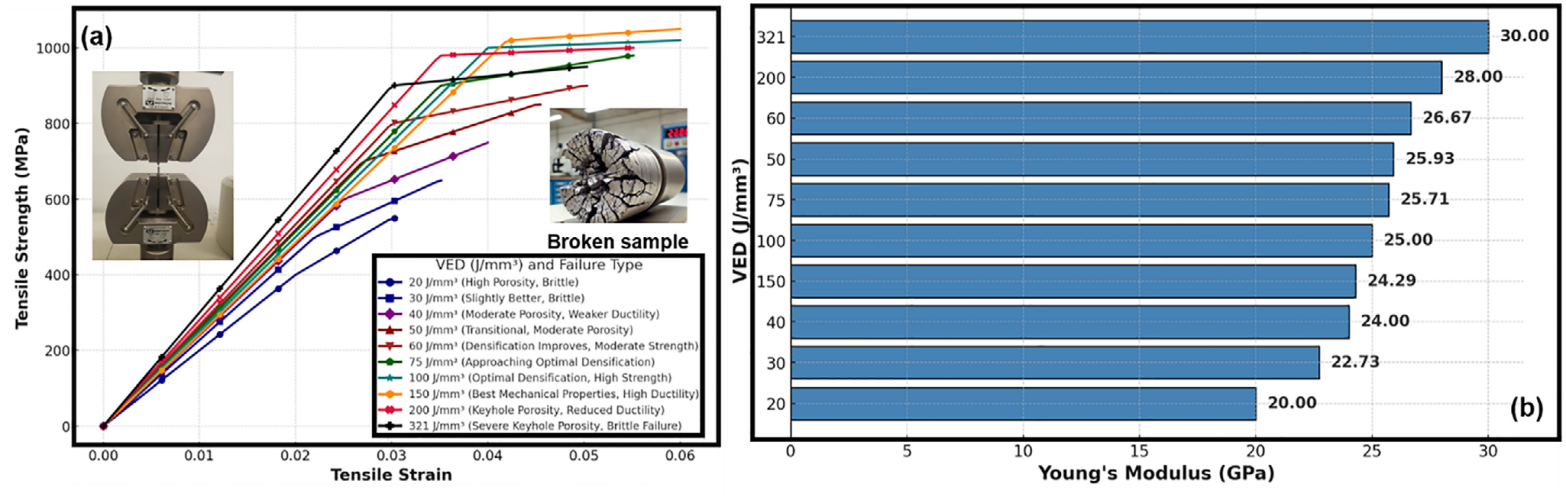
a) Tensile strength versus strain curves for SLM‐fabricated Ti64 samples processed at different Volumetric Energy Densities (VED), illustrating the effect of processing conditions on mechanical performance. The inset images show the tensile test setup and a fractured sample, indicating failure mechanisms. Data are presented as mean ± standard deviation (*n* = 3) for each parameter set. b) Young's Modulus versus VED bar chart, highlighting the variation in elastic modulus with different processing conditions. The results confirm that optimal VED (75–150 J mm^−^^3^) yields the highest strength and modulus, while low and high VED values introduce defects (lack‐of‐fusion and keyhole porosity), reducing mechanical performance. No formal statistical significance testing was applied, as the data represent a mechanical performance trend across processing conditions.

At low VED values (20–50 J mm^−^^3^), the tensile strength and Young's modulus are notably reduced, with the stress–strain curves exhibiting early fracture and minimal elongation as depicted in **Figure** **10**a,b. This behavior directly corresponds to the high porosity fraction and lack‐of‐fusion defects identified in the micro‐CT scans. The incomplete melting due to insufficient energy input results in weak interlayer bonding, making these samples susceptible to premature failure under tensile loading. The presence of large, irregularly shaped voids further exacerbates stress concentration effects, leading to brittle failure. The tensile performance improves significantly as the VED increases to the optimal range (75‐150 J mm^−^^3^). The tensile strength reaches its peak, and the Young's modulus stabilizes at its highest values, i.e., which is consistent with the observed reduction in porosity and improved densification in the micro‐CT analysis. The microstructural evaluation showed a uniform distribution of small, well‐dispersed pores, indicative of a fully consolidated material with minimal defects. This translates into enhanced mechanical stability, higher elongation before fracture, and improved ductility, as evidenced by the more gradual failure observed in the stress–strain curves. The samples fabricated within this range exhibit the most desirable balance between strength and toughness, confirming that this energy density range optimally supports defect‐free material consolidation.

Despite relatively high strength values, a decline in tensile performance is observed at very high VED values (>200 J mm^−^^3^). The stress–strain curves reveal a tendency for sudden failure, attributed to keyhole porosity, as identified in the micro‐CT analysis. The excessive energy input leads to deep vaporization pits that collapse, forming elongated voids and irregular stress concentrators. These defects act as initiation points for crack propagation, reducing ductility and resulting in an inconsistent mechanical response. This trend is particularly evident in the modulus values, which show slight reductions at extreme VEDs, reinforcing the impact of keyhole defects on mechanical integrity.

### 3.2 Healing Kinetics Simulation

#### 3.2.1 Callus Formation Dynamics

A sensitivity analysis was performed to evaluate the influence of boundary condition assumptions on FEA outputs. Four combinations of implant–bone contact (bonded versus frictional) and loading paths (axial versus combined axial‐torsional) were tested. The resulting strain distributions and tissue differentiation patterns were compared, confirming that simulation outcomes remained biologically consistent despite moderate variations in boundary inputs. **Table** **11** provides details of the sensitivity analysis conducted before FEA simulations. A physiological axial compressive load of 700 N was applied to simulate partial weight‐bearing conditions, consistent with prior studies.^[^ ^50^ ^]^ In combined loading scenarios, a torsional moment of 5 Nm was additionally applied to mimic gait‐induced twisting forces.

**Table 11:** Summary of boundary condition sensitivity analysis evaluating the effects of varying implant–bone contact assumptions (bonded vs. frictional) and loading paths (axial vs. combined axial‐torsional) on FEA outcomes. Despite minor shifts in maximum principal strain and strain localization, tissue differentiation patterns and healing timelines remained consistent across all conditions.

Test condition	Max principal strain (Avg)	Peak strain location	Tissue differentiation pattern	Impact on healing timeline	ML prediction robustness
Axial Load + Bonded Contact	0.045	Mid‐diaphyseal cortical region	Endosteal ossification and outer callus formation	No change	High consistency
Axial Load + Frictional Contact	0.047	Slightly shifted toward the metaphyseal edge	Similar pattern, minor changes in callus width	No change	High consistency
Axial + Torsional Load + Bonded Contact	0.052	Increased at the implant‐bone interface	Increased cartilage formation, delayed ossification	Slight delay (1–2 days in ossification onset)	Stable prediction with minor shifts
Axial + Torsional Load + Frictional Contact	0.054	Wider spread along the implant length	Similar to the torsional case, a slightly delayed bone bridge	Slight delay, but healing is still within the physiological window	Stable prediction with minor shifts

**Figure 11:**
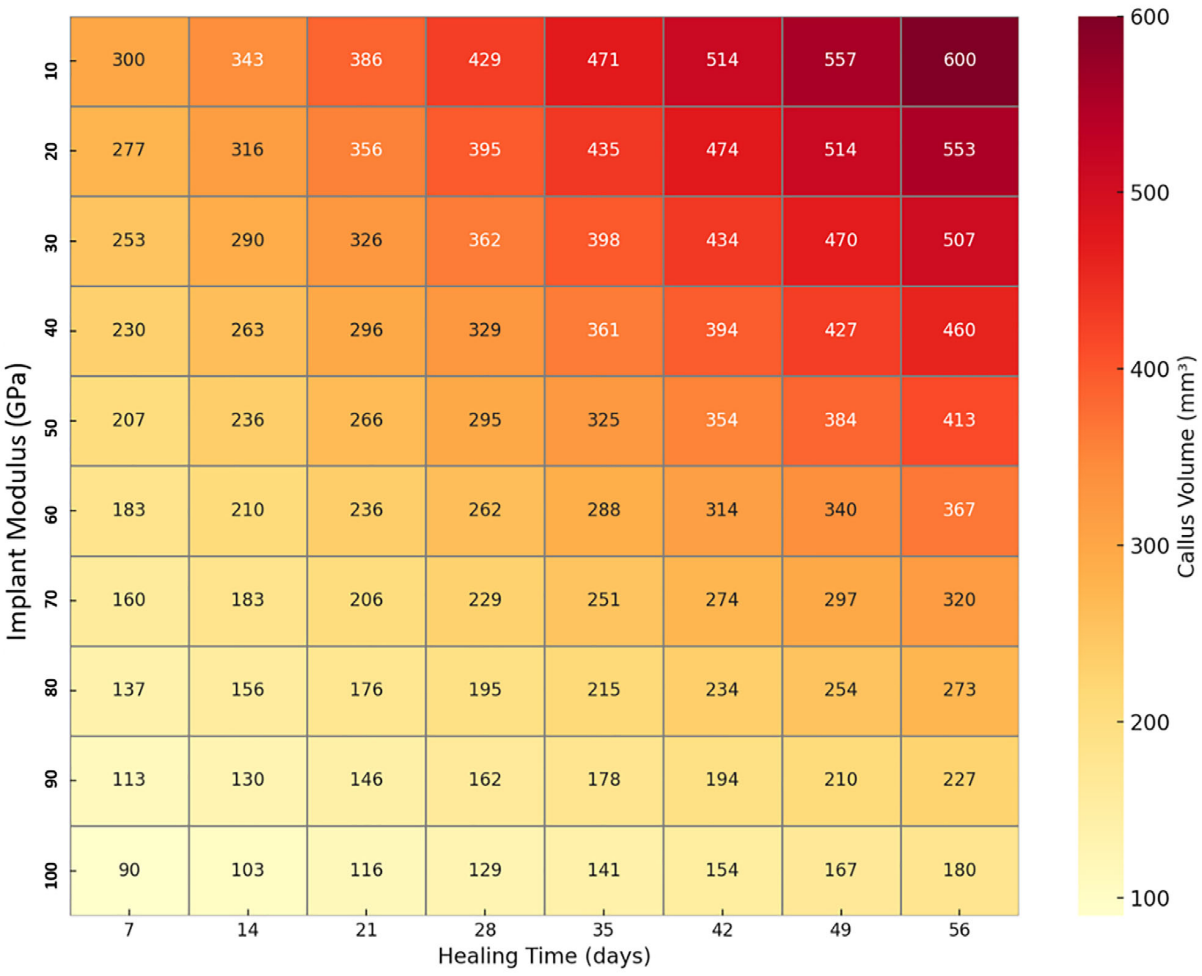
Callus volume evolution over time as a function of implant modulus in a 3 mm transverse tibial fracture model. a) The heatmap illustrates simulated callus volume (in mm^3^) from day 7 to day 56 post‐fracture across a range of implant elastic moduli (2–100 GPa). Lower stiffness implants promote greater callus formation due to increased interfragmentary strain, whereas higher stiffness implants suppress callus development, likely due to stress shielding. Peak callus volumes are observed around day 42 for low‐modulus implants, followed by a plateau phase suggestive of the onset of remodeling. These results underscore the biomechanical influence of implant stiffness on secondary bone healing dynamics.

**Figure** **11** presents a heatmap of the simulated callus volume (mm^3^) as a function of healing time (7 to 56 days) and implant modulus (10 to 100 GPa) for a 3 mm transverse tibial fracture. The data illustrate the interplay between fixation stiffness and biological response during fracture healing.

A clear trend is observed where lower modulus implants (e.g., 10–20 GPa) result in significantly greater callus formation, with volumes reaching up to 600 mm^3^ by day 56. This is attributed to the enhanced interfragmentary strain permitted by more compliant fixation, which stimulates robust periosteal callus development according to mechanoregulation theory. In contrast, higher modulus implants (e.g., 90–100 GPa) consistently show minimal callus formation, not exceeding 180 mm^3^ at any time. This likely results from stress shielding and insufficient mechanical stimulus at the fracture site, which may promote direct (primary) bone healing mechanisms rather than robust callus formation.

Interestingly, the callus volume increases progressively from day 7 to day 42, suggesting active tissue generation. After this period, growth tends to plateau, aligning with the expected transition from callus formation to remodeling. These results emphasize the importance of tailoring implant stiffness to achieve optimal mechanical conditions that favor secondary fracture healing. Moderately stiff implants (e.g., 20–40 GPa) appear to balance stability and mechanical stimulation, resulting in efficient and biologically favorable callus growth.^[^ ^67^ ^]^

### 3.3 Tissue Transformation Progression

The effect of implant stiffness on tibial fracture healing was systematically investigated using a simulation framework grounded in biphasic mechano‐regulation and a cell‐phenotype‐driven callus growth model. A range of implant elastic moduli from 10 to 100 GPa was examined to emulate the stiffness variability achievable through additive manufacturing. Two representative cases, 10 GPa (lowest stiffness) and 100 GPa (highest stiffness), were analyzed in detail to understand the boundary behavior of the healing response, with specific focus on the spatial evolution of mechanical stiffness and bone tissue phenotype concentration within the central (CC) and external callus (EC).

**Figure 12:**
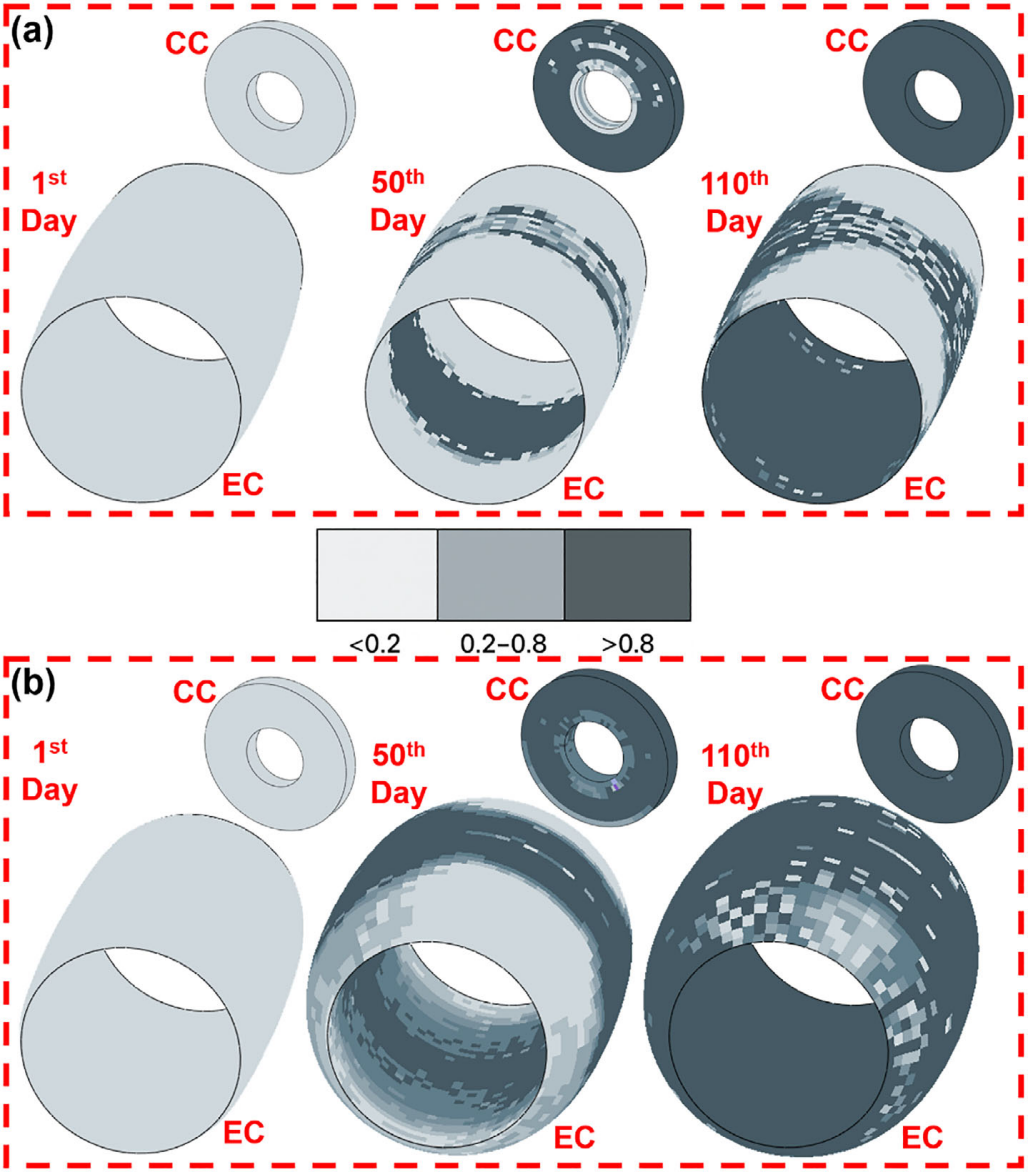
Spatial distribution of bone tissue phenotype concentration in the central callus and external callus at three distinct time points (1st, 50th, and 110th day) for implants with elastic moduli of a) 100 and b) 10 GPa. The grayscale color map indicates normalized bone tissue concentration, where values greater than 0.8 represent mature bone formation. The 10 GPa implant condition b) results in a substantially larger callus volume and more homogeneous bone phenotype development across both CC and EC regions. In contrast, the 100 GPa implant a) exhibits restricted callus formation and delayed tissue development, particularly in the EC, due to the limited mechanical stimulation associated with higher implant stiffness.

**Figure** **12** presents the spatial distribution of bone tissue phenotype concentration across healing days 1, 50, and 110 for both stiffness cases. Figure 12a corresponds to the 100 GPa implant and Figure 12b to the 10 GPa implant. The grayscale scale depicts the normalized concentration of bone tissue phenotype, with values exceeding 0.8 indicating mature bone formation. In the 10 GPa case, extensive callus development is observed, with both CC and EC regions exhibiting progressive and uniform bone tissue formation. By day 110, the phenotype concentration surpasses 0.8 in nearly the entire callus volume. In contrast, the 100 GPa implant results in a markedly smaller callus volume, with the external region exhibiting delayed and spatially limited tissue phenotype development. Central regions in the stiff implant case show early ossification but limited propagation, suggesting that excessive implant rigidity suppresses the mechanical stimuli required for sustained and spatially distributed osteogenesis.^[^ ^49^, ^61^ ^]^

**Figure 13:**
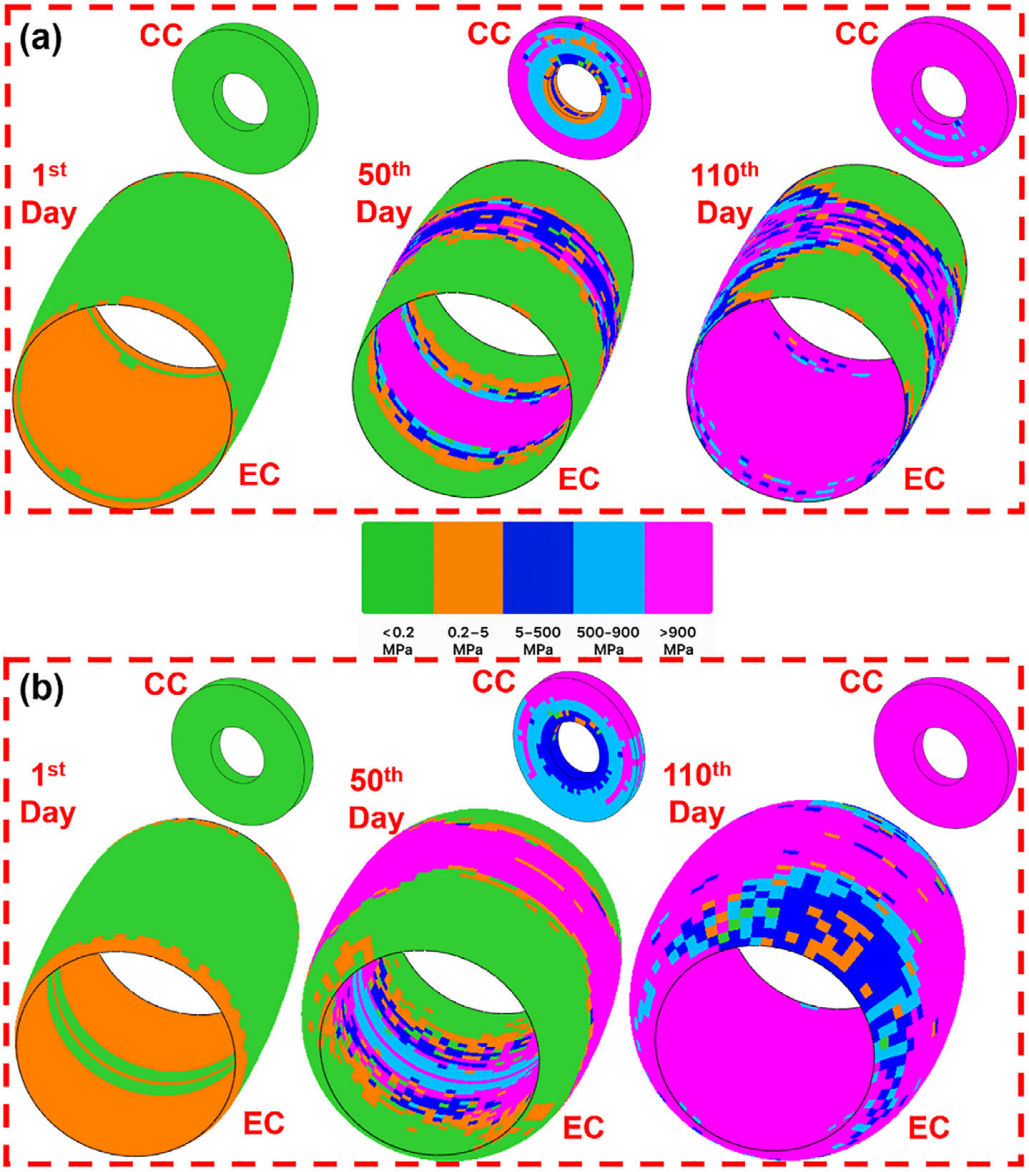
Evolution of callus stiffness (Young's modulus) over the healing period at days 1, 50, and 110 for implants with moduli of a) 100 and b) 10 GPa. The color scale represents local stiffness, ranging from <0.2 MPa (undeveloped tissue) to >900 MPa (fully mineralized bone). The lower‐stiffness implant b) facilitates widespread mechanical reinforcement through progressive mineralization, particularly in the external callus region. In contrast, the stiffer implant a) restricts callus expansion and confines stiffness development primarily to the central callus, highlighting the suppressive effect of high implant rigidity on external callus maturation and spatially distributed healing.

**Figure** **13** shows the evolution of callus stiffness, represented in terms of Young's modulus, over the same time points. Figure 13a reflects the 100 GPa case, while Figure 13b represents the 10 GPa configuration. The colored stiffness scale highlights the progression of tissue modulus, ranging from undeveloped (<0.2 MPa) to fully mineralized bone (>900 MPa). The 10 GPa implant leads to significant volumetric expansion and progressive stiffening of the callus, especially within the EC, where large regions reach values exceeding 900 MPa by day 110. This behavior indicates successful mechanical reinforcement of the healing structure, driven by favorable mechano‐biological stimuli induced by the flexible implant. Conversely, the 100 GPa implant exhibits localized stiffness increases confined to the CC, with the EC remaining largely compliant. This localized ossification and lack of external callus development can be attributed to reduced strain and fluid flow conditions, critical for cell differentiation and matrix mineralization in mechano‐regulation‐driven healing.^[^ ^49^, ^61^ ^]^

The comparative findings between the two stiffness extremes underscore the importance of mechanical compatibility between the implant and the host bone. The 10 GPa implant, whose stiffness approaches that of cortical bone, facilitates strain‐adaptive remodeling and supports endochondral ossification through controlled micromotion. The 100 GPa implant, while providing structural rigidity, suppresses mechanotransduction pathways essential for spatially extensive healing, thereby risking incomplete regeneration or delayed union. These results validate that implant stiffness can be a tunable design parameter for optimizing fracture healing, particularly when leveraged through additive manufacturing processes that allow patient‐specific mechanical tailoring.^[^ ^49^, ^61^ ^]^ In contrast to previous studies that integrate FEA and machine learning primarily for scaffold shape optimization or mechanical property prediction, this study introduces a unified framework that connects SLM process parameters to implant‐level mechanical behavior, simulates biologically validated spatiotemporal bone healing, and leverages AI to enable rapid prediction of healing outcomes. By incorporating a mechanoregulation‐based healing model with empirical in vivo grounding and extending ML capabilities to forecast time‐resolved tissue formation, our approach advances toward clinically meaningful, patient‐specific implant design. While the current study integrates simulation‐based predictions with experimentally derived mechanical properties, we acknowledge the absence of direct biological validation of the healing outcomes. The mechanobiological model used here is based on previously validated frameworks, but further calibration against empirical data for specific implant geometries and patient conditions is necessary. To this end, in vivo and in vitro studies have been planned at a collaborators laboratory to test implants in different bone conditions (e.g., diseased or aged models), which will be used in future studies to refine the model and enhance predictive accuracy.

To further refine the evaluation of optimal stiffness ranges and interpret the healing trajectories across the full design space, AI models were incorporated into the simulation workflow. The following section details the implementation of AI‐based classification techniques used to map stiffness configurations to healing performance outcomes, enabling automated identification of implant designs suited for different fracture types and clinical constraints.

## 4 Artificial Intelligence and Machine Learning Driven Predictive Modeling

### 4.1 Correlation Matrix and Pair Plot

**Figure 14:**
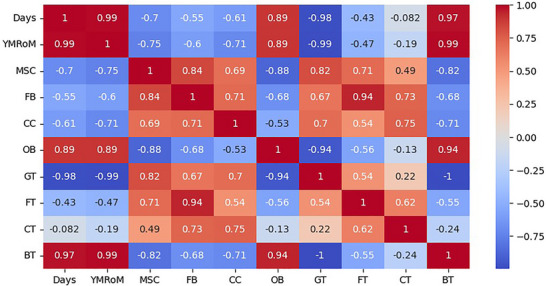
Pearson correlation heatmap illustrating relationships among simulation time (Days), Young's modulus range of the implant (YMRoM), and key cellular and tissue phenotypes, including mesenchymal stem cells, fibroblasts, chondrocytes, osteoblasts, granulation tissue, fibrous tissue, cartilage tissue, and bone tissue. Strong positive correlations are observed between YMRoM and BT (*r* = 0.99), as well as between Days and BT (*r* = 0.97), indicating a consistent progression toward mineralized bone with increased healing time and favorable implant stiffness. Negative correlations between GT and OB/BT reflect the expected transition from immature to mature tissue phenotypes during the healing process. The heatmap provides insight into the interdependence of mechanobiological and phenotypic variables used in the AI‐based classification of healing performance.

The correlation heatmap presented in **Figure** **14** reveals the temporal and mechanistic interdependence between biological tissue formation, cellular responses, and the evolving mechanical properties of the healing environment, as estimated by the Young's Modulus via the Rule of Mixtures (YMRoM). A strong positive correlation (*r* = 0.99) between Days and YMRoM suggests a consistent increase in mechanical stiffness over time, which is in line with the progressive formation of mineralized tissue during bone healing. This mechanical reinforcement is further substantiated by the high positive correlations observed between YMRoM and both osteoblasts (OB, *r* = 0.89) and bone tissue (BT, *r* = 0.99), implying that the emergence and maturation of bone‐forming cells directly contribute to the increase in the composite modulus of the system.

Conversely, a near‐perfect negative correlation between granulation tissue (GT) and YMRoM (*r* = −0.99), as well as between GT and Days (*r* = −0.98), indicates a clear biological transition wherein immature, soft tissue is gradually replaced by stiffer, mineralized bone. This trend underscores the temporal replacement of early‐stage repair tissue with structurally competent bone, a hallmark of successful healing. The strong inverse relationship between GT and BT (*r* = −1.00) further confirms this tissue remodeling process, with bone tissue development progressing at the expense of granulation tissue.

At the cellular level, the matrix also reveals meaningful transitions in cell populations. The MSCs exhibit a strong negative correlation with osteoblasts (*r* = −0.88), suggesting active differentiation and lineage commitment toward bone‐forming phenotypes. Interestingly, fibroblasts and chondrocytes show moderate to strong positive correlations with each other and with osteoblasts, implying that intermediate cell types may transiently support the osteogenic environment before being diminished in the later phases of healing. The mechanical data align with these biological events, with fibroblasts, connective tissue (CT), and fibrous tissue (FT) showing positive correlations with YMRoM, albeit to a lesser extent, indicating their transitional contribution to structural integrity before full mineralization.

Collectively, these correlations illustrate a biologically coherent sequence of events—from stem cell recruitment and soft tissue deposition to osteogenesis and bone maturation—mirrored by a steadily increasing mechanical modulus as quantified by YMRoM. The findings affirm that the rule of mixtures approach effectively captures the dynamic evolution of stiffness within a biologically healing composite system, where tissue‐level transformations directly influence macroscale mechanical behavior. To complement point predictions with uncertainty estimates, prediction intervals were also calculated using quantile regression within the ANN framework. This allowed the model to provide not only accurate forecasts but also a range of likely outcomes, which is particularly valuable for clinical interpretation.

### 4.2 Model Training

The machine learning model training phase commenced with the import of essential Python libraries, including NumPy, Pandas, Matplotlib, Seaborn, Scikit‐learn, and XGBoost, to facilitate data handling, visualization, preprocessing, and model training. Additionally, Keras and TensorFlow were also used later for implementing artificial neural networks. The dataset, comprising ≈1450 datapoints, was derived from mechanoregulation‐based finite element simulations conducted in Abaqus. Each datapoint captured various mechanical, structural, and biological parameters linked to the healing process of bone with SLM‐fabricated lattice implants.

Following data loading, feature selection was conducted to eliminate repetitive and redundant features. This process was guided by domain expertise rather than automated statistical tools. Features with constant values or negligible variance were excluded, as they offered limited contribution to model learning. Additionally, certain variables with unclear biomechanical relevance were discarded to enhance model interpretability and reduce dimensionality. While this approach may not be the most rigorous statistically, it is often used in biomedical applications where domain expertise guides decisions better than purely mathematical rules.

After feature selection, the dataset was examined for missing or anomalous entries, particularly within the target variables: “Days,” “YMROM” (Young's Modulus Rate of Mixture), and “Modulus.” These inconsistencies were primarily attributed to numerical instabilities or computational constraints during simulation runs. Instead of applying conventional imputation techniques such as mean, median, or median replacement, the Iterative Imputer from Scikit‐learn was employed. This method iteratively estimates missing values using Random Forest Regression, a non‐parametric and ensemble‐based technique that leverages relationships among multiple features. The iterative approach improves the accuracy of imputed values by preserving underlying correlations and non‐linear patterns.

The dataset was then partitioned into independent (X) and dependent (y) variables. The X matrix included all input parameters, while the y vector comprised the three regression targets: “Days,” “YMROM”, and “Modulus”. This separation was critical for defining supervised learning objectives. To evaluate model generalizability, the dataset was randomly split into training and testing subsets using an 80/20 ratio. This stratified partitioning ensures unbiased performance evaluation by preventing information leakage from the training to the test set. Such a split allows quantification of model performance on unseen data and is a standard protocol in regression‐based predictive modeling.

Given the absence of categorical variables in the dataset—all features being continuous or numerical—no encoding was performed. Encoding techniques (e.g., one‐hot, label encoding) are generally employed to convert non‐numerical categories into a numerical format compatible with machine learning algorithms. However, in this case, all features were inherently numerical and biologically quantitative (e.g., modulus, strain, surface area), negating the requirement for such transformation. Before training, standardization was applied using the StandardScaler utility from Scikit‐learn. This method transforms each feature to have zero mean and unit variance, ensuring that all input variables are on a comparable scale. Importantly, standardization was applied after the train‐test split to prevent data leakage from the training set into the test set. This is a critical step in preserving the integrity of the evaluation process, as applying standardization before splitting could inadvertently introduce information from the test set into the training process, leading to overly optimistic performance estimates. Standardization is particularly important for algorithms sensitive to feature magnitude, such as support vector machines and artificial neural networks. It facilitates improved convergence of optimization algorithms and enhances predictive accuracy by mitigating scale‐induced biases.

With preprocessing complete, multiple machine learning algorithms were trained and evaluated as given in **Table** **12**. These included ensemble regressors such as Random Forest, XGBoost, and Gradient Boosting, selected for their robustness against overfitting and ability to model non‐linear relationships inherent in biomechanical data. In addition to ensemble models, Artificial Neural Networks (ANNs) were implemented using Keras with TensorFlow backend. A feedforward neural network architecture was designed with multiple hidden layers, ReLU activation functions, and an output layer with linear activation to accommodate continuous regression outputs. The model was compiled using the Adam optimizer and trained with early stopping to prevent overfitting. ANNs are particularly advantageous in capturing complex interactions among biomechanical parameters, especially where explicit modeling of dependencies is not feasible. Model performance was quantified using regression metrics such as Mean Squared Error (MSE), Root Mean Squared Error (RMSE), and the coefficient of determination (R^2^ score). MSE measures the average squared difference between the predicted and actual values, providing insight into the variance of the errors. MAE calculates the average magnitude of the errors without considering their direction, offering an intuitive measure of prediction accuracy. The R^2^ score indicates the proportion of variance in the target variable explained by the model, with values closer to one signifying stronger explanatory power. These metrics allowed for a comprehensive assessment and comparison of the models' performance, facilitating the selection of the most suitable algorithm for the specific application.

**Table 12:** Regression algorithms results.

S.No	Model	MSE	MAE	R^2^ Score
0	XGBoost Regression	18.497621	2.438068	0.988804
1	Random Forest Regression	27.705801	3.226684	0.981414
2	Gradient Boosting	52.097071	4.581939	0.973871
3	Decision Tree Regression	57.730238	4.503848	0.963164
4	KNN Regression	108.820407	5.657754	0.914455
5	Polynomial Regression	220.681878	9.394839	0.787493
6	Linear Regression	410.517425	15.326930	0.730208
7	Ridge Regression	624.586627	19.400179	0.669897
8	Lasso Regression	793.169799	22.482618	0.626743
9	Support Vector Regression	4424.672471	37.387513	0.590469
10	Elastic Net Regression	12395.820990	67.558242	0.259123

The application of machine learning to simulate bone healing trajectories using mechanobiological data led to promising outcomes, particularly when regressive predictive modeling was employed. Among the eleven regression algorithms tested on the dataset derived from finite element simulations, three models exhibited outstanding predictive accuracy in terms of the R^2^ metric. These were XGBoost Regression, Random Forest Regression, and Gradient Boosting Regression, with R^2^ values reaching 98.88%, 98.14%, and 97.38%, respectively. These results underscore the robustness of ensemble learning methods when applied to complex biomedical data involving nonlinear relationships between microstructural lattice features and healing parameters such as time, tissue strain, and modulus. The high scores achieved by these three models made them the most reliable predictors within the scope of our study. **Table** **13** provides details of actual and predicted values when the model is trained by the XGBoost algorithm.

**Table 13:** Difference between actual and predicted values when the model is trained on the XGBoost algorithm.

Sr. No.	Actual days	Predicted days	Difference days	Actual YMRoM	Predicted YMRoM	Difference YMRoM	Actual Young modulus	Predicted Young modulus	Difference Young modulus
1.	23	22.316	0.684	119.887	122.322	−2.436	70	70.063	−0.063
2.	31	29.178	1.822	181.647	176.740	4.907	50	49.978	0.022
3.	16	17.253	−1.253	86.052	86.558	−0.507	90	81.237	8.763
4.	33	32.818	0.182	231.092	232.478	−1.386	20	20.836	−0.836
5.	24	25.675	−1.675	197.977	198.036	−0.059	15	15.379	−0.379
6.	66	65.486	0.514	256.281	256.016	0.265	80	89.090	−9.090
7.	108	109.821	−1.821	848.076	848.895	−0.819	15	14.996	0.004
8.	52	50.961	1.039	252.405	256.545	−4.140	40	40.236	−0.236
9.	12	12.487	−0.487	40.496	47.726	−7.231	60	60.069	−0.069
10.	27	26.126	0.874	142.204	141.680	0.525	80	88.571	−8.571
11.	71	71.925	−0.925	305.973	305.395	0.577	90	80.597	9.403
12.	66	66.179	−0.179	278.641	270.452	8.188	50	76.266	−26.266
13.	86	85.445	0.555	629.011	643.975	−14.964	15	14.949	0.051
14.	85	86.166	−1.166	508.736	505.443	3.293	70	72.884	−2.884
15.	85	84.924	0.076	579.228	583.145	−3.917	20	19.569	0.431
16.	8	7.997	0.003	11.545	11.330	0.215	20	19.910	0.090
17.	47	46.558	0.442	222.659	222.021	0.639	70	69.995	0.005
18.	32	29.760	2.240	196.735	189.635	7.100	40	39.842	0.158
19.	50	50.088	−0.088	280.533	279.564	0.970	25	29.458	−4.458
20.	28	29.028	−1.028	294.389	310.141	−15.752	5	4.955	0.045
21.	1	1.005	−0.005	0.200	0.246	−0.046	50	41.370	8.630
22.	69	68.965	0.035	335.539	335.888	−0.348	25	29.990	−4.990
23	86	85.957	0.043	596.046	595.842	0.204	20	19.982	0.018
24.	44	44.104	−0.104	271.184	271.395	−0.211	30	25.216	4.784
25.	67	67.417	−0.417	318.941	322.627	−3.686	25	25.858	−0.858
26.	19	19.508	−0.508	99.339	112.454	−13.116	90	83.754	6.246
27.	29	28.283	0.717	152.588	150.118	2.470	80	88.930	−8.930
28.	58	57.042	0.958	228.720	228.729	−0.009	80	89.409	−9.409
29	50	49.732	0.268	398.465	399.297	−0.832	10	10.102	−0.102

A deeper examination of why these models performed better than others can be attributed to the computational architecture and learning paradigms they adopt. XGBoost, or Extreme Gradient Boosting, is a highly optimized implementation of gradient boosting algorithms that incorporates several enhancements, such as regularization (both L1 and L2) and parallelized tree construction.^[^ ^68^ ^]^ It constructs additive decision trees sequentially, where each successive tree corrects the residuals of the previous ones, allowing the model to iteratively refine its predictions. This reduces both bias and variance, enabling the model to generalize better without overfitting. In contrast to simpler models like linear regression or decision trees, XGBoost's regularization mechanisms control model complexity while maintaining strong predictive capabilities, especially when dealing with noisy or irregular data, which is common in biomedical simulations.^[^ ^69^ ^]^

Random Forest Regression, the second‐best performing algorithm, operates using a different ensemble strategy known as bagging, or bootstrap aggregating (random sampling of a dataset with replacement). Here, multiple decision trees are trained independently on different bootstrapped samples of the data. The final prediction is then averaged across all trees, resulting in a robust and stable estimator. Random Forest tends to perform well on high‐dimensional data and can capture complex nonlinear interactions without requiring intensive parameter tuning.^[^ ^70^ ^]^ Gradient Boosting, which also builds models in a sequential manner like XGBoost, operates on the principle of minimizing a loss function by constructing weak learners stage by stage. This partly explains why its R^2^ value was slightly lower, although still competitive. All three methods benefited from their ability to uncover deep and complex interactions among features without requiring manual feature transformations, making them well‐suited for tasks like ours, where biological and mechanical variables interact in non‐trivial ways.

While model architecture played a central role in achieving high predictive performance, preprocessing and feature selection also influenced the results to a limited extent. Feature engineering, carried out based on domain expertise rather than statistical automation, involved removing irrelevant or constant‐value columns that could otherwise introduce noise. Although no categorical variables were present in the dataset, making one‐hot encoding unnecessary, standardization was still applied post train‐test split to avoid data leakage. Interestingly, standardization had only a marginal impact on model performance, as tree‐based models are relatively insensitive to feature scaling. Nevertheless, these steps added a layer of precaution to ensure clean input and prevent biased learning.^[^ ^71^ ^]^

An important implication of these findings lies in how machine learning can facilitate inverse design strategies for biomedical implants. With these predictive models, it becomes feasible to reverse‐engineer implant properties by predicting the biological outcomes, such as the healing time or the modulus of regenerated tissue, by specifying the cell and tissue percentage amounts or ratios, and determining the optimal lattice configurations or mechanical conditions needed to achieve them. **Table** **14** demonstrates that the predictive model closely approximated the actual values of healing time, YMRoM, and Young's Modulus, with minor deviations across most test samples, highlighting the model's generalization capability and its potential for clinical application in personalized healing assessment. This dramatically reduces the need for repetitive simulations and trial‐based physical prototyping, enabling data‐driven personalization in bone implant manufacturing. Such capabilities highlight how machine learning can act as a surrogate modeling tool that enhances both the efficiency and intelligence of design workflows in biomedical engineering.^[^ ^72^ ^]^

**Table 14:** Comparison between actual and predicted values for healing days, YMRoM, and Young's Modulus, along with their corresponding differences for 29 test samples when using XGBoost Regression ML Algorithm.

Sr. No.	Actual days	Predicted Days	Difference days	Actual YMRoM	Predicted YMRoM	Difference YMRoM	Actual Young modulus	Predicted Young modulus	Difference Young modulus
1.	23	22.316	0.684	119.887	122.322	−2.436	70	70.063	−0.063
2.	31	29.178	1.822	181.647	176.740	4.907	50	49.978	0.022
3.	16	17.253	−1.253	86.052	86.558	−0.507	90	81.237	8.763
4.	33	32.818	0.182	231.092	232.478	−1.386	20	20.836	−0.836
5.	24	25.675	−1.675	197.977	198.036	−0.059	15	15.379	−0.379
6.	66	65.486	0.514	256.281	256.016	0.265	80	89.090	−9.090
7.	108	109.821	−1.821	848.076	848.895	−0.819	15	14.996	0.004
8.	52	50.961	1.039	252.405	256.545	−4.140	40	40.236	−0.236
9.	12	12.487	−0.487	40.496	47.726	−7.231	60	60.069	−0.069
10.	27	26.126	0.874	142.204	141.680	0.525	80	88.571	−8.571
11.	71	71.925	−0.925	305.973	305.395	0.577	90	80.597	9.403
12.	66	66.179	−0.179	278.641	270.452	8.188	50	76.266	−26.266
13.	86	85.445	0.555	629.011	643.975	−14.964	15	14.949	0.051
14.	85	86.166	−1.166	508.736	505.443	3.293	70	72.884	−2.884
15.	85	84.924	0.076	579.228	583.145	−3.917	20	19.569	0.431
16.	8	7.997	0.003	11.545	11.330	0.215	20	19.910	0.090
17.	47	46.558	0.442	222.659	222.021	0.639	70	69.995	0.005
18.	32	29.760	2.240	196.735	189.635	7.100	40	39.842	0.158
19.	50	50.088	−0.088	280.533	279.564	0.970	25	29.458	−4.458
20.	28	29.028	−1.028	294.389	310.141	−15.752	5	4.955	0.045
21.	1	1.005	−0.005	0.200	0.246	−0.046	50	41.370	8.630
22.	69	68.965	0.035	335.539	335.888	−0.348	25	29.990	−4.990
23	86	85.957	0.043	596.046	595.842	0.204	20	19.982	0.018
24.	44	44.104	−0.104	271.184	271.395	−0.211	30	25.216	4.784
25.	67	67.417	−0.417	318.941	322.627	−3.686	25	25.858	−0.858
26.	19	19.508	−0.508	99.339	112.454	−13.116	90	83.754	6.246
27.	29	28.283	0.717	152.588	150.118	2.470	80	88.930	−8.930
28.	58	57.042	0.958	228.720	228.729	−0.009	80	89.409	−9.409
29	50	49.732	0.268	398.465	399.297	−0.832	10	10.102	−0.102

Despite the promising performance of the best algorithms, several limitations persist, which are largely inherent to data‐driven approaches in biomedical applications. One of the primary challenges in this study was the relatively limited size of the dataset, consisting of just over 1400 entries. Although ensemble models can handle small‐to‐moderate data volumes better than some other algorithms, predictive accuracy and generalizability would certainly improve with larger and more diverse datasets. More data would allow the models to capture subtle interactions between input features and outcomes, especially in edge cases where the biological variability is significant. Additionally, the dataset was derived from simulated mechanoregulation models rather than clinical or in vivo data, which may introduce simulation‐specific biases that limit the transferability of the trained models to real‐world scenarios.

Another important consideration is the interpretability of these regression models. While ensemble methods such as XGBoost and Random Forest offer feature importance scores, the underlying decision pathways are complex and often opaque, leading to what is commonly referred to as the “black box” nature of machine learning.^[^ ^73^ ^]^ This lack of transparency can be problematic, particularly in clinical settings where decisions based on model predictions must be well justified. Though these models demonstrate strong predictive power, they do not inherently provide mechanistic explanations for why certain lattice configurations result in specific healing outcomes. This epistemological gap poses a limitation for researchers who aim to blend mechanistic modeling with data‐driven insights in a holistic framework.

Ensemble regression algorithms proved effective in predicting tissue regeneration parameters based on lattice design and loading conditions. The top‐performing models—XGBoost, Random Forest, and Gradient Boosting—achieved high accuracy and demonstrated robustness in handling complex, high‐dimensional data. While preprocessing steps like standardization and feature refinement had a limited impact on performance, they helped ensure data integrity and prevent leakage. These models show potential for streamlining inverse design workflows, though future research should address concerns around data sufficiency and model interpretability. Overall, the results emphasize the role of machine learning in biomedical engineering, particularly for tasks that benefit from predictive accuracy in experimental planning and personalized treatment strategies.

### 4.3 Discussion of Artificial Neural Networks Results

Artificial neural networks have emerged as one of the most transformative innovations in the field of machine learning, drawing inspiration from the structure and functioning of the human brain. Mimicking the biological neurons and synapses, ANNs consist of interconnected layers of nodes that communicate through weighted connections, dynamically adjusting during training to minimize prediction error.^[^ ^74^ ^]^ Their ability to model non‐linear relationships, adapt to complex datasets, and generalize across varying inputs has led to a surge of interest across multiple disciplines. From image and speech recognition to natural language processing, drug discovery, and personalized medicine, ANNs are powering advancements that were previously inconceivable with traditional algorithms. Their appeal lies not only in their predictive accuracy but also in their flexibility—ANNs can scale up with data volume and complexity, making them an indispensable tool in domains that require data‐driven insights from intricate systems.^[^ ^75^ ^]^

In this study, we implemented artificial neural networks using TensorFlow to investigate the predictive potential of ANNs in modeling outcomes related to bone implant simulations. The dataset used for training consisted of 1450 data points generated through finite element simulations, capturing variations in Young's modulus and healing time. To ensure consistency, a batch size of 32 was maintained across all model trainings, while the architectural parameters were varied systematically. Specifically, the number of hidden layers, the number of neurons per layer, and the number of training epochs were incrementally modified to assess their impact on the model's performance, as evident in **Table** **15**. The goal was to identify optimal configurations that could yield high predictive accuracy, as measured by the R^2^ score, which serves as an indicator of how well the predicted values align with the actual outcomes. Through this systematic experimentation, we sought to uncover the interaction between architectural complexity and training duration, aiming to balance model expressiveness with computational efficiency.

**Table 15:** Performance metrics (MSE, MAE, and R^2^ Score) of Artificial Neural Network (ANN) models with varying architectures and training epochs for predicting healing outcomes.

S.No	ANN Layers & Neurons	Epochs	MSE	MAE	R^2^ Score
	Layer 1 = 32	1000	860.27	22.82	0.62
		2000	516.07	17.72	0.65
	Layer 1 = 64	1000	739.52	20.87	0.64
		2000	456.18	16.37	0.66
	Layer 1 = 128	1000	567.36	18.43	0.65
		2000	366.33	14.45	0.70
	Layer 1 = 256	1000	430.72	15.73	0.67
		2000	326.68	13.38	0.73
	Layer 1 = 512	1000	389.06	14.78	0.69
		2000	328.19	13.49	0.75
	Layer 1 = 32 Layer 2 = 64	1000	275.47	11.70	0.78
		2000	118.86	6.90	0.88
	Layer 1 = 64 Layer 2 = 128	1000	134.57	7.05	0.87
		2000	53.52	4.37	0.95
	Layer 1 = 128 Layer 2 = 256	1000	65.64	4.93	0.93
		2000	39.19	3.51	0.96
	Layer 1 = 256 Layer 2 = 512	1000	81.25	5.81	0.92
		2000	37.03	3.29	0.96
	Layer 1 = 512 Layer 2 = 1024	1000	42.71	3.83	0.96
		2000	33.12	3.27	0.97
	Layer 1 = 32 Layer 2 = 64 Layer 3 = 128	1000	130.52	7.53	0.90
		2000	58.58	4.94	0.95
		3000	37.43	3.46	0.96
	Layer 1 = 64 Layer 2 = 128 Layer 3 = 256	1000	77.57	5.72	0.93
		2000	40.91	3.60	0.96
		3000	29.96	3.02	0.97
	Layer 1 = 128 Layer 2 = 256 Layer 3 = 512	1000	40.57	3.68	0.96
		2000	29.11	3.39	0.97
		3000	20.03	2.32	0.98
		4000	19.23	2.20	0.98
	Layer 1 = 256 Layer 2 = 512 Layer 3 = 1024	1000	51.26	4.64	0.95
		2000	33.48	3.19	0.97
		3000	28.45	2.89	0.97
		4000	21.74	2.36	0.98
	Layer 1 = 512 Layer 2 = 512 Layer 3 = 512	1000	44.86	3.83	0.95
		2000	22.02	2.50	0.98
		3000	28.20	2.79	0.97
		4000	20.78	2.27	0.97
		5000	25.88	2.78	0.97
	Layer 1 = 1024 Layer 2 = 1024 Layer 3 = 1024	1000	70.32	5.71	0.96
		2000	26.48	2.99	0.97
		3000	21.66	2.41	0.97
		4000	19.50	2.43	0.98
		5000	15.87	1.78	0.98

Our analysis highlighted clear trends in how neural network architecture impacts model performance. With a single hidden layer, increasing the number of neurons led to gradual improvements in R^2^ score—from 0.62 at 32 neurons to 0.75 at 512 neurons—though the gains became marginal beyond 128 neurons, suggesting a point of diminishing returns. This improvement is likely due to the model's enhanced ability to capture complex relationships in the data. However, the most significant leap in accuracy came with the shift to deeper, two‐layer networks. For example, a model with 64 and 128 neurons across two layers reached an R^2^ of 0.95 at 2000 epochs—significantly outperforming the best single‐layer setup. This underscores how deeper architectures help uncover more abstract and meaningful patterns, thereby improving prediction quality.

Furthermore, extending the architecture to three hidden layers pushed the performance boundaries even further. Notably, the combination of 128, 256, and 512 neurons across three layers, trained for 4000 epochs, delivered the best R^2^ score of 0.98. This configuration consistently outperformed other combinations, even those with higher neuron counts or more epochs, suggesting that an optimal balance of depth, width, and training duration is key to maximizing model generalization. The relatively high R^2^ score in this case is likely due to the network's ability to model intricate dependencies in the data, made possible by its layered structure and extended training time.^[^ ^76^ ^]^ Importantly, simply increasing the number of neurons or epochs beyond this point did not yield better results. For instance, models with 1024 neurons in all three layers or those trained for more than 4000 epochs did not consistently surpass the R^2^ score achieved by the 128–256–512 configuration. The ANN model with three hidden layers (128–256–512 neurons) performed very well, closely matching the actual values for healing days, YMRoM, and Young's modulus as evident in **Table** **16**. In most cases, the prediction errors were quite small and within a reasonable range, showing that the model can be trusted for practical use.

**Table 16:** Comparison between actual and predicted values of healing time (Days), YMRoM, and Young's Modulus using the best‐performing ANN model (Layer 1 = 128, Layer 2 = 256, Layer 3 = 512 at 4000 epochs).

S.No	Actual Days	Predicted Days	Difference Days	Actual YMRoM	Predicted YMRoM	Difference YMRoM	Actual Young Modulus	Predicted Young Modulus	Difference Young Modulus
1	26	26.392	−0.392	185.657	191.157	−5.500	20	22.099	−2.099
2	107	110.160	−3.160	812.949	819.920	−6.971	30	30.928	−0.928
3	104	107.106	−3.106	712.306	717.060	−4.754	90	88.300	1.700
4	85	85.337	−0.337	498.735	500.374	−1.639	80	93.383	−13.383
5	69	69.895	−0.895	294.452	295.608	−1.156	60	63.580	−3.580
6	68	68.744	−0.744	275.990	275.924	0.066	70	80.825	−10.825
7	2	2.111	−0.111	0.200	0.458	−0.258	50	51.292	−1.292
8	76	76.990	−0.990	434.483	436.322	−1.839	20	28.828	−8.828
9	46	46.568	−0.568	273.988	276.095	−2.107	25	27.469	−2.469
10	79	78.866	0.134	460.872	464.077	−3.205	30	26.840	3.160
11	63	62.609	0.391	227.127	235.805	−8.678	100	96.727	3.273
12	73	74.099	−1.099	344.974	346.718	−1.744	40	44.483	−4.483
13	24	24.129	−0.129	126.812	130.322	−3.510	70	71.208	−1.208
14	102	104.240	−2.240	672.144	681.463	−9.319	100	107.851	−7.851
15	95	94.447	0.553	614.116	612.455	1.661	100	104.101	−4.101
16	45	45.936	−0.936	435.910	440.692	−4.782	5	5.102	−0.102
17	64	64.531	−0.531	428.305	430.915	−2.610	10	11.471	−1.471
18	51	51.110	−0.110	279.620	281.729	−2.108	30	25.662	4.338
19	8	7.313	0.687	11.440	12.182	−0.742	30	27.554	2.446
20	85	85.899	−0.899	668.830	671.155	−2.325	10	12.816	−2.816
21	43	43.494	−0.494	242.042	248.431	−6.389	40	40.577	−0.577
22	101	103.499	−2.499	801.163	805.456	−4.293	15	18.254	−3.254
23	92	93.445	−1.445	611.835	618.384	−6.549	70	79.438	−9.438
24	71	71.483	−0.483	564.029	566.663	−2.634	5	6.432	−1.432
25	45	46.582	−1.582	215.741	216.880	−1.139	90	85.379	4.621
26	19	19.330	−0.330	109.565	109.030	0.534	60	58.751	1.249
27	60	60.698	−0.698	284.832	280.988	3.844	30	29.344	0.656
28	100	102.069	−2.069	726.811	730.190	−3.379	40	47.776	−7.776
29	14	14.586	−0.586	58.426	60.880	−2.454	80	85.415	−5.415
30	54	53.951	0.049	242.878	242.940	−0.062	60	60.692	−0.692

In addition to point estimates, the model's prediction intervals were also computed using quantile regression, providing a 90% confidence range around the outputs. The average interval widths were narrow, 1.90 for Days, 9.14 for YMRoM, and 7.33 for Modulus, indicating both high certainty and practical reliability in the model's predictions. Despite the promising results obtained through ANN training, the performance was still bound by certain limitations. One of the primary challenges encountered was the limited size of the dataset. Although 1450 data points provided a reasonable basis for initial training, deep neural networks generally require large volumes of high‐quality data to fully realize their potential. The data sparsity constrained the model's ability to generalize and may have led to fluctuations in performance across configurations.^[^ ^77^ ^]^ This limitation was particularly evident in the earlier epochs and in shallower networks, where the R^2^ scores remained low despite increased complexity. With more extensive datasets, particularly those incorporating broader clinical or biomechanical scenarios, the model could be expected to learn more nuanced patterns and demonstrate better predictive stability.

One of the key challenges in using neural networks is their “black box” nature—despite their high accuracy, it's often unclear how the model arrives at its predictions. This lack of interpretability makes it difficult to understand why certain configurations perform better or to extract meaningful insights from the model's behavior. In biomedical applications, such as predicting healing outcomes or optimizing implant designs, this limitation can affect trust in AI‐generated decisions unless supported by explainable AI methods or sensitivity analysis.^[^ ^78^ ^]^ Additionally, overfitting is a common risk, particularly in deep architectures trained over extended epochs. Without enough diverse and high‐quality input data, models may capture noise or spurious patterns, leading to inflated training performance without reliable real‐world accuracy.

Our results highlight the importance of carefully selecting neural network parameters—such as the number of layers, neurons per layer, and training epochs—to achieve reliable performance. While increasing model depth and training time generally improved accuracy, there was a clear point beyond which further complexity offered minimal benefits and sometimes reduced generalization. The best‐performing setup used three hidden layers with 128, 256, and 512 neurons and was trained for 4000 epochs, reaching an R^2^ score of 0.98. This emphasizes the need for thoughtful experimentation and tuning when applying neural networks to biomechanical prediction tasks.

## 5 Conclusion

This work establishes a technically rigorous, integrative approach to optimizing orthopedic implants by coupling experimental SLM fabrication, mechanobiological simulation, and AI‐driven prediction. Fabrication of Ti64 specimens under varying SLM process parameters revealed that porosity and mechanical strength are highly sensitive to VED. Micro‐CT and mechanical testing showed that VED values between 75 and 150 J mm^−^^3^ result in optimal densification, minimizing both lack‐of‐fusion and keyhole defects, while providing adequate tensile and elastic performance. These experimental insights were fed into a high‐resolution finite element model of tibial fracture healing, which used a mechano‐regulatory algorithm incorporating strain, pore pressure, and interstitial fluid velocity to simulate callus growth, cell differentiation, and tissue maturation.

A parametric study was performed on implants with stiffnesses ranging from 10 to 100 GPa, emulating solid and lattice configurations achievable through additive manufacturing. Implants at the lower end of this stiffness spectrum significantly enhanced external callus expansion and triggered sustained endochondral ossification, as evidenced by spatially distributed tissue phenotype transitions and increased callus modulus. In contrast, higher‐stiffness implants limited micromechanical stimulus and promoted centralized, less robust healing pathways. These biomechanical responses were quantitatively validated through simulation of cell population dynamics, mineralization progression, and stiffness evolution over time.

To eliminate reliance on computationally intensive simulations, an AI‐based predictive framework was developed using supervised regression algorithms trained on the simulation dataset. The resulting models enabled accurate prediction of healing performance metrics, such as bone tissue volume and callus stiffness, based on implant design parameters. This hybrid methodology allows for real‐time, patient‐specific optimization of implant architecture and mechanical behavior, facilitating the translation of SLM‐enabled, lattice‐based implants into clinically adaptive solutions.

## Conflict of Interest

The authors declare no conflict of interest.

## Author Contributions

M.U.Z. contributed to conceptualization, visualization, data curation, image design, methodology, wrote, reviewed & edited the final manuscript, and also contributed to writing the original draft and revision. M.H.R. contributed to the visualization, Methodology, and reviewed, investigated & edited the final project. M.F.A. contributed to conceptualization, visualization, supervision, wrote, reviewed & edited the final manuscript, acquired funding and resources, and also contributed to writing the original draft and revision. Y.K.M. contributed to conceptualization, visualization, supervision, investigation, wrote, reviewed & edited the final manuscript, acquired funding and resources, and also contributed to writing the original draft and revision.

## Data Availability

The data that support the findings of this study are available from the corresponding author upon reasonable request.
